# Defined Electrosynthetic Microbial Consortia Reveal Electron Transfer Modes Governing Acetate Production

**DOI:** 10.1002/advs.202513340

**Published:** 2025-11-14

**Authors:** Jing Zhang, He Liu, Qihao Cao, Chao Zhang, Min Zhang, Minhua Cui, Yan Zhang, Bo Fu, Hongbo Liu

**Affiliations:** ^1^ School of Environment and Ecology Jiangnan University Wuxi 214122 China; ^2^ Jiangsu Collaborative Innovation Center of Water Treatment Technology and Material Suzhou 215011 China

**Keywords:** acetogens, carbon dioxide conversion, microbial electrosynthesis, *shewanella oneidensis* MR‐1, synthetic microbial consortia

## Abstract

Precise manipulation of interspecies electron transfer (IET) is critical for advancing microbial electrosynthesis (MES) toward efficient CO_2_ bioconversion. Here, defined synthetic consortia are constructed by pairing *Shewanella oneidensis* MR‐1, a model bidirectional electroactive bacterium, with *Clostridium aceticum* (electroactive acetogen) or *Acetobacterium woodii* (nonelectroactive acetogen), mimicking functional guilds commonly observed in natural MES communities. Co‐cultivation markedly boosts acetate production by up to 88%, achieving 1.16 ± 0.01 and 1.05 ± 0.01 g L^−1^, with carbon conversion efficiencies exceeding 84%. Comprehensive electrochemical, spectroscopic, and biomass analyses reveal distinct spatial electron transfer modes: DIET via cytochrome *c* and riboflavin dominates at the biofilm‐electrode interface in *S. oneidensis–C. aceticum* consortia, whereas *S. oneidensis–A. woodii* consortia prefer H_2_/formate‐mediated IET in the planktonic phase. This metabolic stratification enables *S. oneidensis* to function as an “ecosystem engineer,” orchestrating electron flow to optimize CO_2_‐to‐acetate conversion across biofilm and suspension niches. The proposed synthetic ecology strategy provides a blueprint for designing high‐efficiency MES consortia, paving new avenues for sustainable carbon capture and bio‐based chemical production.

## Graphical abstract

Spatially resolved Shewanella–acetogen interactions link DIET and MET pathways to acetate formation, offering a generalizable framework for designing structured, high‐efficiency microbial electrosynthesis systems.

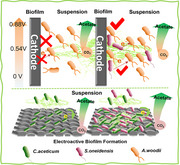

## 1 Introduction

Biological conversion of CO_2_ into chemicals and fuels is one of the key strategies to mitigate climate change and advance a circular carbon economy. Microbial electrosynthesis (MES) couples renewable electricity with microbial metabolism to drive CO_2_ reduction into value‐added products such as acetate and alcohols, offering a sustainable alternative to thermochemical routes.^[^ ^1^ ^]^ Mixed microbial consortia are often applied in MES due to their metabolic flexibility and operational robustness. However, their inherent heterogeneities, such as unstable composition and complex interspecies interactions, introduce challenges for process control and obscures mechanistic interpretation.^[^ ^2^ ^]^ Thus, clarifying community dynamics and resolving electron transfer mechanisms among functional groups remains a central task for MES optimization.^[^ ^3^ ^]^

Interspecies electron transfer (IET) is fundamental to MES performance because it determines how cathodic electrons are distributed among community members. Two major IET modes have been established: direct interspecies electron transfer (DIET), based by conductive cytochromes or *pili*, and mediated electron transfer (MET), which depends on diffusible carriers such as H_2_, formate, or flavins.^[^ ^4^ ^]^ Open MES communities typically enrich both exoelectrogens (e.g., *Geobacter spp*., *Shewanella spp*.), electroactive acetogens (e.g., *Clostridium ljungdahlii*), and non‐electroactive acetogens (e.g., *Acetobacterium spp*.).^[^ ^2^, ^3^ ^]^ This coexistence suggests that DIET and MET operate simultaneously, yet it remains difficult to assign specific functional roles to each taxon.^[^ ^2^, ^5^ ^]^ Although genomic and pure‐culture studies have revealed intracellular energy conservation pathways of several acetogens,^[^ ^6^, ^7^, ^8^ ^]^ they cannot explain how spatial organization (biofilm versus planktonic niches) drives partitioning and coupling of electron flow.^[^ ^9^ ^]^

Defined microbial consortia provide a tractable approach to bridge this gap.^[^ ^10^ ^]^ In this study, we constructed synthetic consortia comprising *Shewanella oneidensis* MR‐1, *Clostridium aceticum*, or *Acetobacterium woodii* to elucidate IET pathways and their spatial‐functional organization. *S. oneidensis* MR‐1 is notable for its efficient extracellular electron transfer (EET) through cytochromes (MtrC, OmcA) and flavin secretion.^[^ ^5^, ^11^ ^]^ *C. aceticum* has been reported to exhibit electroactive traits,^[^ ^12^, ^13^ ^]^ while *A. woodii* cannot directly accept electrode‐derived electrons^[^ ^7^ ^]^ but can interact with electroactive partners via diffusible mediators.^[^ ^1^ ^]^ Yet, the extent of *S. oneidensis* MR‐1′s bidirectional electron transfer in co‐cultures and its regulatory role in product synthesis remain unclear.

Therefore, this study aims to: 1) systematically quantify how *S. oneidensis* MR‐1 reorganizes product formation when paired with electroactive versus non‐electroactive acetogens under identical cathodic conditions, and 2) mechanistically resolve how electrode‐attached biofilms and planktonic fractions partition IET to govern CO_2_‐to‐acetate flux. To achieve these aims, we integrated complementary approaches, including electrochemical measurements (DPV peak potentials, EIS charge‐transfer resistance), metabolite profiling (H_2_, formate, riboflavin), biomass distribution and *fhs* gene copy numbers, and functional prediction. These convergent datasets distinguish biofilm‐localized DIET from mediator‐driven MET and link electron transfer routes with spatial product distribution. Collectively, this framework advances mechanistic understanding of defined consortia and provides guidance for designing high‐efficiency MES systems for sustainable CO_2_ bioconversion.

## 2 Results and Discussion

### 2.1 Co‐Culture Enhances Acetate Synthesis

**Figure 1:**
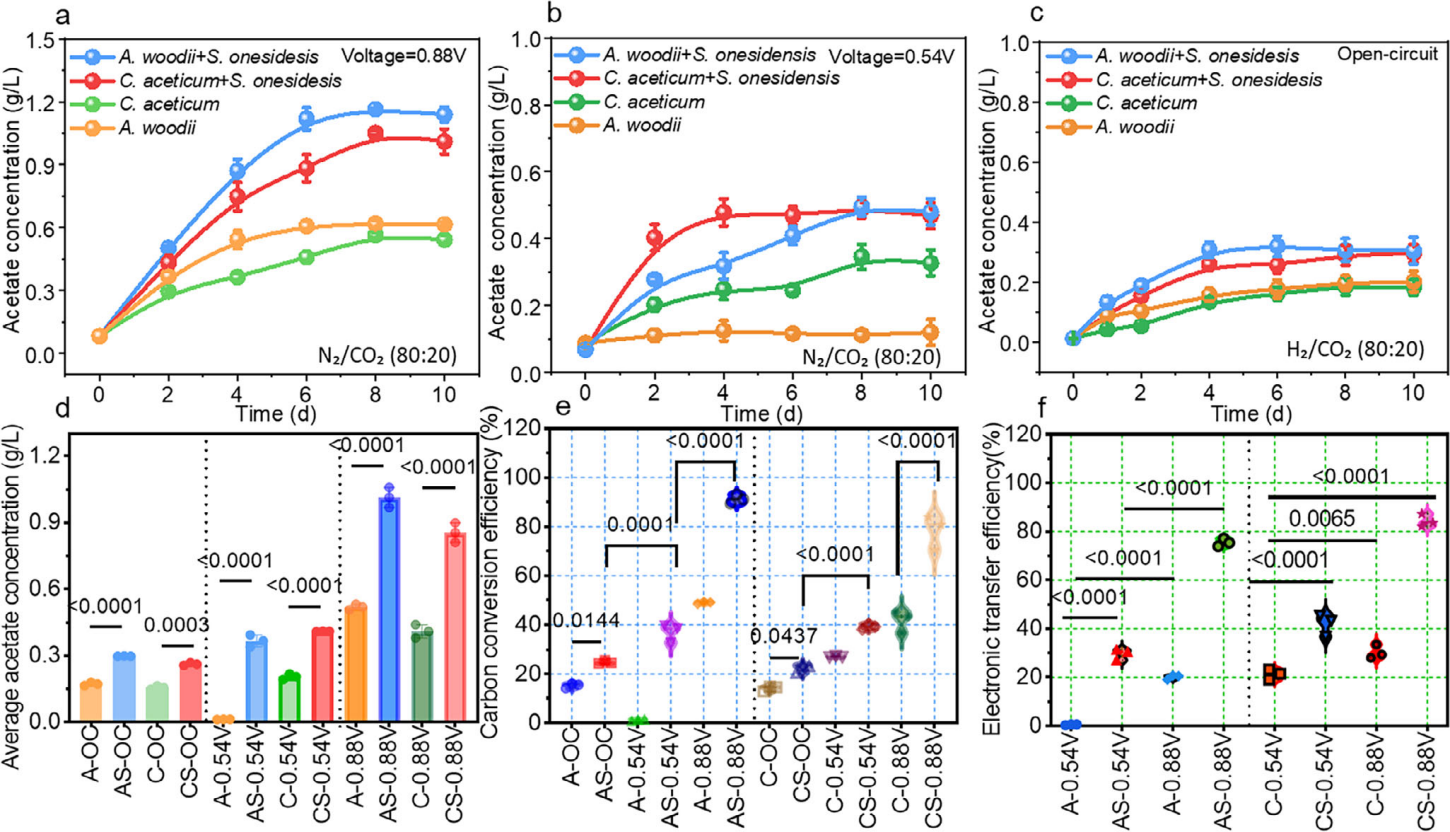
Overview of acetate production and system performance across different culture strategies and electrochemical conditions a) Product synthesis performance in monoculture and co‐culture systems under applied voltage of 0.88 V; b) Performance under applied voltage of 0.54 V; c) Performance under open‐circuit conditions; d) Average acetate production after system stabilization; e) Carbon conversion efficiency; and f) Electron transfer efficiency.

**Figure** **1** presents the acetate synthesis and electron transfer efficiency (ETE) of different mono‐ and co‐cultures in MES systems under varying voltage conditions. Under an applied voltage of 0.88 V, acetate accumulation in monoculture MES systems reached 0.62 ± 0.04 g L^−1^ for the nonelectroactive acetogen *A. woodii* and 0.56 ± 0.04 g L^−1^ for the electroactive acetogen *C. aceticum* (Figure 1a). In the co‐culture AS system (*A. woodii* + *S. oneidensis* MR‐1), acetate concentration increased to 1.16 ± 0.01 g L^−1^, representing a 1.87‐fold of *A. woodii* alone. Similarly, in the co‐culture CS system (*C. aceticum* + *S. oneidensis* MR‐1), acetate accumulation reached 1.05 ± 0.01 g L^−1^, 1.88 times of that in *C. aceticum* monoculture. The corresponding carbon conversion efficiency (CCE) of the AS and CS system were 93.12% ± 0.56% and 84.03% ± 1.01%, respectively (Figure 1e), while ETE was improved to 75.58% ± 1.61% and 84.40% ± 2.63% (Figure 1f). Notably, the AS system exhibited a preferable synergistic effect on acetate synthesis, whereas the CS system demonstrated superior electron transfer efficiency, highlighting the strain‐specific differences in electron transfer and resource allocation.

Under an applied voltage of 0.54 V, the co‐culture AS and CS systems also exhibited significantly enhanced acetate synthesis compared to monocultures. Under this condition, non‐electroactive acetogen *A. woodii* alone was unable to directly take electrons from the electrode and failed to produce acetate (Figure 1b). However, the co‐culture AS system markedly enhanced acetate synthesis to 0.50 ± 0.03 g L^−1^, suggesting that *S. oneidensis* MR‐1 facilitated *A. woodii* for electrosynthesis by MET. In contrast, since *C. aceticum* was capable of accepting external electrode electrons for acetate production even in the absence of hydrogen as an electron donor, the maximum acetate concentration reached 0.35 ± 0.04 g L^−1^. The co‐culture CS system further increased acetate accumulation to 0.49 ± 0.03 g L^−1^ (Figure 1b). Correspondingly, the CCE of co‐culture AS and CS systems improved by 31.52% ± 3.10% and 11.44% ± 5.23%, respectively (Figure 1e), while ETE increased by 29.11% ± 2.15% and 20.53% ± 5.53% (Figure 1f).

Notably, AS and CS consortia exhibited different acetate production profiles under varying cathodic potentials. Under an applied voltage of 0.88 V, the AS consortium achieved the highest acetate concentration, whereas the CS consortium showed a better acetate concentration under an applied voltage of 0.54 V, with both systems ultimately reaching similar product performances. These differences may reflect the varying capacities of *A. woodii* and *C. aceticum* to access and utilize H_2_ and electrode‐derived electrons. *A. woodii* may perform more efficiently under limited H_2_ availability, while *C. aceticum* benefits more from increased H_2_ production or direct electron uptake at lower electrode potentials. This may be due to the use of HDCR complexes by *A.woodii* for CO_2_ fixation,^[^ ^14^ ^]^ where H_2_ dependent CO_2_ reductases may be more advantageous. A benchmark comparison with representative MES studies (Table S1, Supporting Information) highlights the quantitative positioning of our system. The achieved acetate titer (1.16 ± 0.01 g L^−1^), CCE (>84%), and ETE (>75%) are within or above the range of prior reports operated.^[^ ^15^, ^16^ ^]^ This confirms that the defined‐consortia framework developed here is quantitatively competitive with established MES systems.

The open‐circuit experiment results demonstrated that even without an applied voltage, the co‐culture with *S. oneidensis* MR‐1 enhanced acetate production for both *A. woodii* and *C. aceticum* (Figure 1c). Given that microbial metabolites such as riboflavin, formate, and hydrogen can act as electron shuttles,^[^ ^4^, ^11^ ^]^ acetate synthesis likely involves not only electron exchange between the cathode and cathodic biofilm but also electron transfer between the cathode and planktonic microbial communities through these shuttles. Additionally, at potentials above the hydrogen evolution threshold, co‐cultures exhibited greater CO_2_ conversion efficiency, presumably due to cathodic hydrogen acting as an electron donor that supports both biofilm‐associated and planktonic microbial metabolism. *S. oneidensis* MR‐1 appears to facilitate *C. aceticum* for acetate production through direct electron transfer and the secretion of electron mediators (e.g., riboflavin), which enhances electron exchange between the electrode and suspension. Collectively, these findings demonstrate that across all tested conditions, *S. oneidensis* MR‐1 consistently enhances acetate synthesis in co‐culture via DIET and MET through electron shuttles.

Additionally, 3D‐EEM analysis (Figure S1, Supporting Information) reveals that under all tested conditions, the fluorescence intensity in quadrant IV of the co‐culture system is significantly enhanced, indicating that co‐culturing with *S. oneidensis* MR‐1 boosts the overall metabolic activity of the microorganisms. The fluorescence signal of riboflavin (E_x_/E_m_ = 470/520 nm) is notably stronger, suggesting its role as an electron mediator in the cathodic electron transfer process. Furthermore, the NADH fluorescence peak (E_x_/E_m_ = 340/400 nm) is more prominent in the co‐culture system compared to the monoculture system, implying that *S. oneidensis* MR‐1 enhances the intracellular supply of reducing power (NADH), thus facilitating CO_2_ conversion and acetate synthesis.

### 2.2 IET Mechanism in the Co‐Culture Consortia

**Figure 2:**
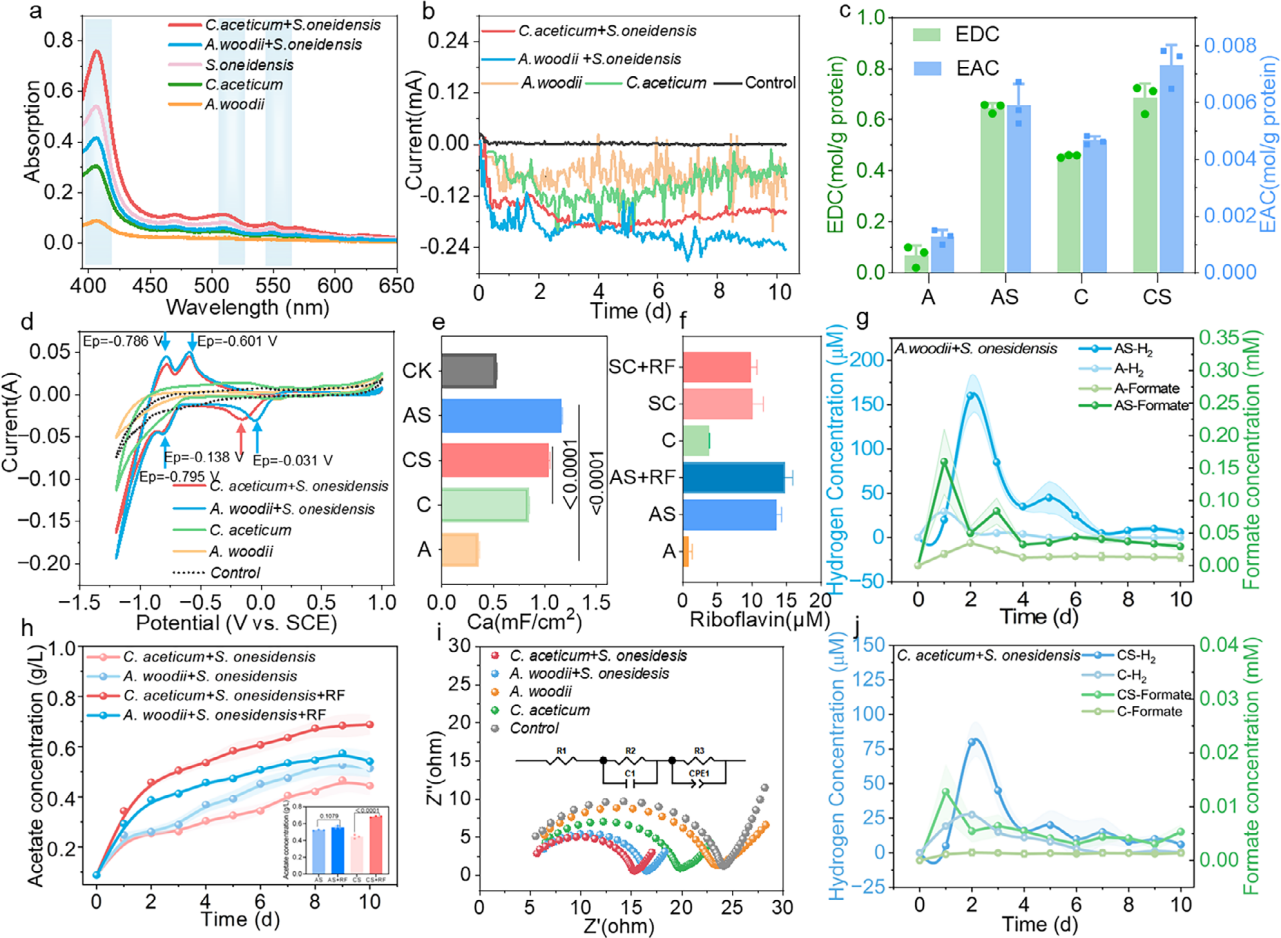
Electrochemical characterization and metabolite profiles of co‐culture consortia under applied voltage of 0.88 V. a) UV–vis spectra of cytochrome *c* in the co‐culture consortia; b) Current variation in the co‐culture consortia; c) Electron acceptance and donation capacities (EAC and EDC) in different consortia. d) CV curves of CS single‐species pure culture and two‐species co‐culture consortia. e) Area capacitance in bio‐cathode CVs. f) riboflavin content in the consortia. g) Hydrogen production and formate variation in AS single‐species pure culture and two‐species co‐culture consortia. h) Effect of riboflavin addition on acetate production in AS and CS consortia. i) EIS of the consortia. j) Hydrogen production and formate variation in CS single‐species pure culture and two‐species co‐culture consortia. Note: To enhance clarity, one representative phase from the three replicates is shown. And the curve of cathode potential variation over time is shown in the supplementary material Figure S2 (Supporting Information).

Compared to monoculture, the characteristic absorption peaks of cytochrome c (410, 530, and 565 nm)^[^ ^17^ ^]^ were significantly enhanced in the CS co‐culture consortia (**Figure** **2**a), indicating that the DIET between *S. oneidensis* MR‐1 and *C. aceticum* was intensified. However, in the AS consortia, the cytochrome C content was lower than in the *S. oneidensis* MR‐1 monoculture, suggesting that IET might occur through membrane permeability regulation or the use of endogenous electron shuttles. The riboflavin fluorescence signal (E_x_/E_m_ = 470/520 nm) in the co‐culture consortia was notably higher (Figure S1, Supporting Information), particularly in the CS consortia, where riboflavin secretion was especially prominent (Figure 2f), reinforcing its role as a key mediator in IET.

**Figure 3:**
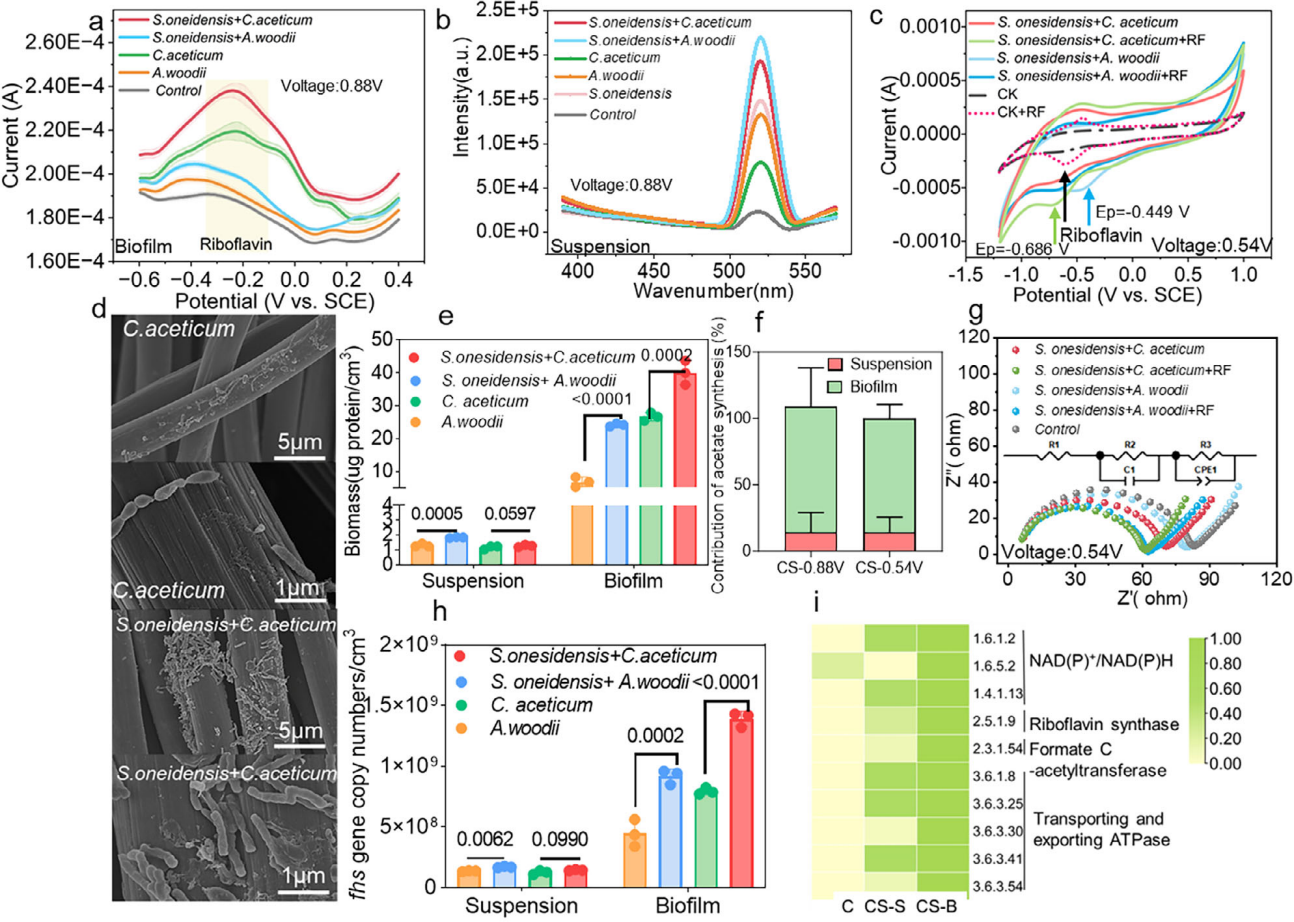
Spatial differentiation and electrochemical behavior in the *C. aceticum–S. oneidensis* MR‐1(CS) electrosynthetic consortium. a) DPV curves of the cathode biofilm and b) full fluorescence spectra of the cathode suspension under applied voltage of 0.88 V; c) CV curves of MES with and without riboflavin under applied voltage of 0.54 V; d) formation of the electrode biofilm in *C. aceticum* monoculture and the CS co‐culture consortia under applied voltage of 0.88 V; e) biomass content (as measured by protein content) of the cathode biofilm and suspension formed by monoculture and co‐culture strains; f) contribution rates of planktonic microorganisms and biofilms in the CS consortia to acetate production under applied voltage of 0.88 and 0.54 V g) EIS impedance; h) *fhs* gene copy number per unit volume in each consortia; i) expression of key functional enzymes in the electrode biofilm and suspension of *C.aceticum* monoculture and co‐culture consortia.

To assess riboflavin's role, we added it exogenously. In the AS consortia, acetate increased slightly from 0.50 ± 0.02 to 0.54 ± 0.03 g L^−1^ (a 9.07% ± 5.58% increase), but the change was not significant (*p* > 0.05) (Figure 2h), indicating that other synergistic pathways may be involved. In contrast, the acetate titer in the CS+RF consortia significantly reached 0.69 ± 0.04 g L^−1^ (increased by 40.70% ± 4.08%) (Figure 2h), highlighting the importance of riboflavin in facilitating electron transfer and product synthesis between *S. oneidensis* MR‐1 and electroactive *C. aceticum*. In addition to riboflavin‐mediated IET, hydrogen production was significantly higher in both AS and CS cocultures compared to monocultures (Figure 2g,j). Previous studies have shown that *S. oneidensis* MR‐1 can enhance cathodic hydrogen evolution,^[^ ^18^ ^]^ thereby supporting mediator‐assisted electron transfer that ultimately promoted acetate synthesis in both consortia. Notably, the AS consortium exhibited slightly higher current consumption than the CS consortium (Figure 2b), likely because *A. woodii* relies more strictly on hydrogen as an electron donor, requiring additional current for hydrogen generation. This indicates that *S. oneidensis* enhanced the metabolism of *A. woodii* through hydrogen evolution, while in *A. woodii*, the hydrogen‐dependent CO_2_ reductase (HDCR) can utilize H_2_ to reduce CO_2_ into formate, thereby fueling the methyl branch of the Wood–Ljungdahl pathway and enhancing acetate formation.^[^ ^19^ ^]^ Furthermore, the cathodes in the co‐culture AS and CS consortia exhibited significantly higher EAC and EDC than those in the monoculture (Figure 2c), along with preferable electrocatalytic activity (Figure S3, Supporting Information). This was further supported by Tafel slope (𝛽) analysis, where lower 𝛽 values corresponded to enhanced electron transfer and electrocatalytic efficiency.^[^ ^20^ ^]^ The electrochemical activity of the AS consortia was particularly higher (Figure S3, Supporting Information), which may be attributed to the more effective synergy between *A. woodii* and *S. oneidensis* MR‐1. Additionally, higher conductivity and more negative redox potential (Figure 3c; Figure S3a, Supporting Information) suggest that the electron transfers in the co‐culture consortia are more effective.

Cyclic voltammetry (CV) analysis of the co‐culture AS and CS consortia (Figure 2d) revealed a reversible redox peak at ≈−0.32 and −0.37 V, attributed to the electron conversion of flavin‐bound MtrC/OmcA.^[^ ^21^, ^22^ ^]^ The significantly higher peak currents in the co‐cultures indicate an accelerated reaction rate and improved electrosynthesis efficiency. Notably, the negative shift in the conversion peak potential suggests an increased availability of hydrogen and formate, potentially facilitating a transition from one‐step to multi‐step electron transfer processes. This shift correlates with higher current consumption, highlighting the critical roles of hydrogen production and formate conversion in the metabolic process. Moreover, the co‐culture consortia exhibited a substantial increase in capacitance (1.12 mF cm^−^^2^ for AS and 1.16 mF cm^−^^2^ for CS, Figure 2e), likely due to the enrichment of electroactive compounds within the electrode biofilm, thereby enhancing electron storage and transfer capacity.

EIS analysis provides further insight into the mechanism underlying the enhanced ETE in the co‐culture consortia (Figure 2i). In the CS consortia, the biofilm resistance (R2) and charge transfer resistance (R3) decreased by 48.81% and 42.08%, respectively, compared to monoculture of *C. aceticum* (Table S2, Supporting Information), indicating that *S. oneidensis* MR‐1 promotes the formation of electroactive biofilms and reduces electron transfer resistance. Similarly, in the AS consortia, the solution resistance (R1), biofilm resistance (R2), and charge transfer resistance (R3) were reduced by 17.22%, 9.63%, and 37.80%, respectively, compared to the monoculture of *A. woodii*, further confirming the superior electron transfer performance of the co‐culture consortia. These improvements are closely associated with the increased secretion of EPS (Figure S3b,f). While EPS promotes electron transfer, excessive secretion could increase impedance. Thus, the dynamic balance between EPS secretion and composition is crucial for optimizing electron transfer in the co‐culture consortia. This further validates that the co‐culture consortia enhance ETE and electrocatalytic activity by optimizing cathode impedance and EPS secretion.

### 2.3 Biofilm and Suspended Cell Characterization in Electroactive Bacterial Co‐Cultures

**Figure 4:**
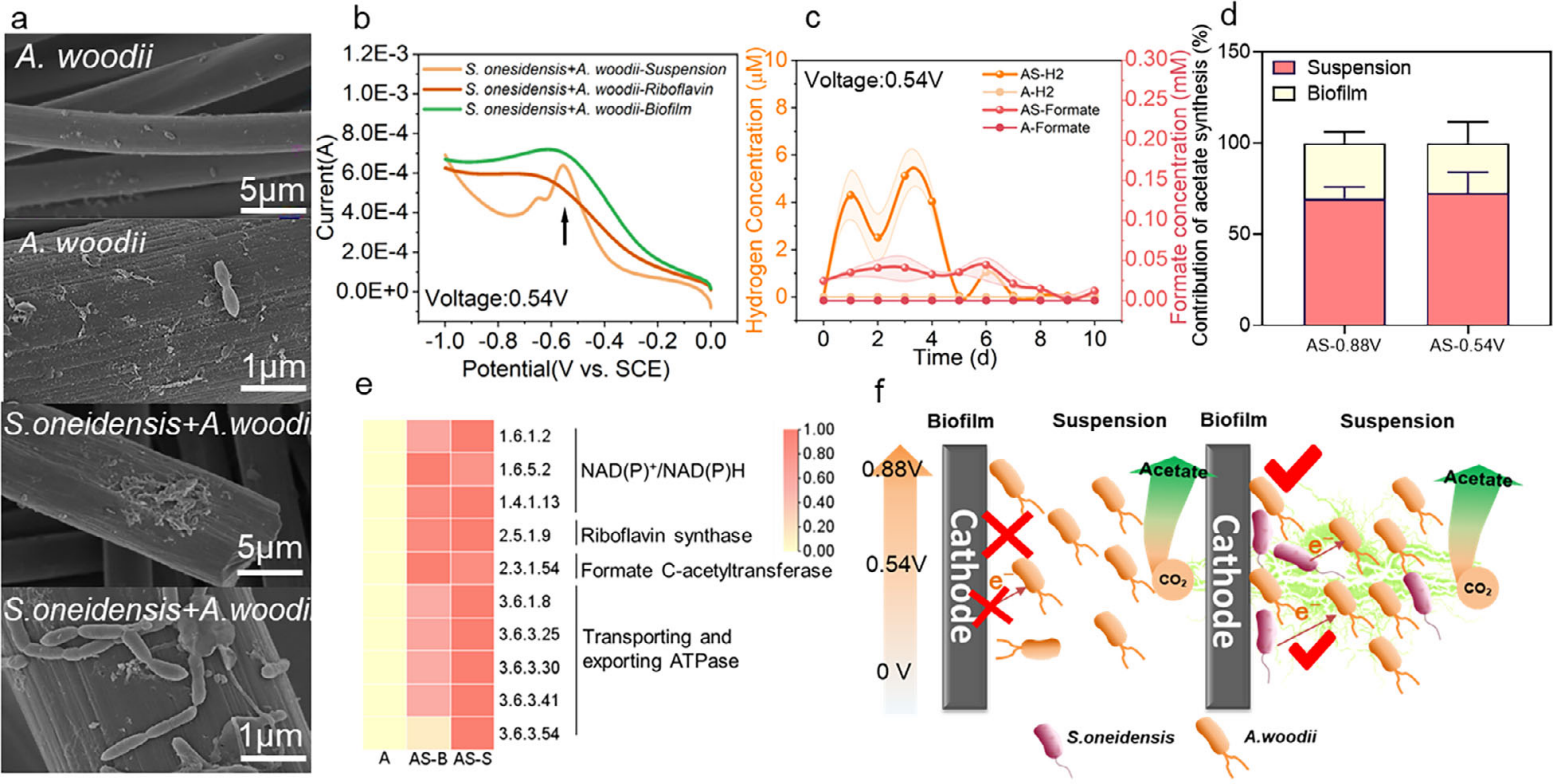
Electron transfer and product synthesis mechanisms in the *A. woodii–S. oneidensis* MR‐1(AS) electrosynthetic consortium. a) Electrode biofilm formation of *A.woodii* alone and co‐cultured AS consortia above the hydrogen precipitation potential; b) LSV curves of biofilms and suspensions of the co‐cultured AS consortia; c) hydrogen and formate variations of the *A.woodii* alone and AS consortia; d) the contribution of the planktonic‐state microorganisms and biofilm contribution to acetic acid synthesis in the MES system; e) expression of relevant functional enzymes in the electrode biofilm and suspension of *A.woodii* alone and co‐cultured AS; f) mechanism of product synthesis in the electrode biofilm and suspension in the AS co‐culture consortia.

The primary electron transfer mechanism of *S. oneidensis* MR‐1 involves the use of soluble riboflavin or flavin mononucleotide as electron shuttles, contributing ≈70–90% of the electron transfer process.^[^ ^23^, ^24^ ^]^ The cathode biofilm community is rich in riboflavin, with a characteristic oxidation peak observed at −0.244 V vs SCE.^[^ ^25^ ^]^ Notably, the biofilm in the CS consortia exhibits the highest riboflavin content (**Figure** **3**a), suggesting that its electron transfer primarily occurs at the biofilm interface. In contrast, the riboflavin signal in the biofilm of the AS consortia is weak, but a significant riboflavin fluorescence peak (520 nm) is observed in the suspension (Figure 3b), indicating that its electron transfer is dependent on free riboflavin. These findings are further supported by electrochemical tests, where the CS+RF consortia showed the best electrochemical performance, with the highest unit capacitance of the cathode biofilm (Figure 3c; Figure S4a, Supporting Information) and the lowest impedance (Figure 3g), along with superior electrocatalytic performance (Figure S3h, Supporting Information) and consistent with its product synthesis efficiency (Figure 2h). Below the hydrogen‐evolution potential (HEP), LSV analysis reveals that the riboflavin characteristic peak (−0.6 V vs SCE) in the AS consortia is located in the planktonic microorganisms (Figure 4b), whereas in the CS consortia, it is located at the biofilm interface (Figure S4b, Supporting Information). As illustrated in Figure 3d, while *C. aceticum* alone formed a sparse biofilm on the electrode, co‐cultivation led to a substantial increase in cathodic biofilm density (Figure 3e), indicating enhanced microbial metabolic activity. Furthermore, the biomass (protein measurement) on the electrode in the co‐culture CS and AS was measured at 40.04 ± 5.19 and 24.17 ± 0.65 µg cm^−3^, respectively, significantly higher than the monoculture groups at 26.68 ± 1.76 and 12.38 ± 18.19 µg cm^−3^. Meanwhile, the biomass in the suspension of the co‐culture AS consortia was 1.84 ± 0.65 µg cm^−3^, higher than the monoculture value of 1.25 ± 0.17 µg cm^−3^, whereas the biomass in the CS consortia suspension (1.28 ± 0.19 µg cm^−3^) was nearly identical to the monoculture (1.23 ± 0.48 µg cm^−3^). Quantitative biomass analysis indicated that the CS consortia, through the synergistic interaction of *S. oneidensis* MR‐1 and *C. aceticum*, greatly promoted microbial growth within the biofilm (increased *fhs* gene copy number, Figure 3h), while the AS consortia increased biomass in both the biofilm and suspension, suggesting that *A. woodii* is more dependent on free‐floating microbial metabolites. Additionally, *C. aceticum* exhibited significantly lower capacitance on the bioelectrode compared to other co‐culture combinations, particularly above the HEP (Figure S4a, Supporting Information), indicating that *C. aceticum* primarily relies on surface growth rather than suspension growth. This also suggests that *S. oneidensis*'s contribution to *C. aceticum* promotion is primarily evident in the biofilm rather than the suspension. Moreover, substantial differences were observed in the expression of functional genes between the biofilm and planktonic organisms. Compared to monocultures, enzymes associated with NAD(P)H synthesis, cytochrome *c*‐related enzymes (key components of the electron transport chain), enzymes involved in the Wood‐Ljungdahl pathway, and ATPase transport enzymes in the CS biofilm (CS‐B) were significantly upregulated, with much greater expression levels than those in the suspension (CS‐S) (Figure 3i). Combining all the above observations, it is deduced that acetate synthesis is likely concentrated within the biofilm in the CS consortia.

To verify this hypothesis, we periodically removed planktonic microorganisms under conditions below and above the HEP. Results showed that below the HEP, the cathodic biofilm in the CS co‐culture accounted for a higher proportion of acetate production (70.46–127.06%), a trend that persisted even above the HEP (77.14–97.56%). The decline in the biofilm's relative contribution at higher potentials may be attributed to the facilitative effect of hydrogen diffusion on acetate production by planktonic cells, as hydrogen was occasionally detected in the consortia (Figure S4c, Supporting Information). Furthermore, interestingly, we found that *S. oneidensis* MR‐1 can grow using acetate as the sole carbon source, which may suggest that *S. oneidensis* MR‐1 can accelerate the fixation process of CO_2_ by acetogens by relieving product accumulation (Figure S5, Supporting Information).

The metabolic interaction between *S. oneidensis* MR‐1 and *C. aceticum* revealed a synergistic “acetate‐formate cycle.” *S. oneidensis* MR‐1 assimilates acetate secreted by *C. aceticum* for growth and ATP synthesis, mitigating the risk of product inhibition from acetate accumulation. Concurrently, *S. oneidensis* MR‐1 generates formate, which serves as a substrate for the methyl branch of the WLP in *C. aceticum*, promoting CO_2_ fixation and acetate synthesis. This cycle enhances consortia performance through multiple mechanisms: 1) Formate and riboflavin act as soluble electron shuttles, reducing the interfacial charge transfer resistance (R_ct_ decreased by over 40%); 2) Spatial metabolic division minimizes competition for intermediates, leading to a 1.78‐fold increase in acetate concentration; 3) ATP generated from acetate metabolism drives riboflavin secretion, further enhancing the DIET between *S. oneidensis* MR‐1 and *C. aceticum*, establishing a stable and efficient cooperative metabolic network. Additionally, the increased hydrogen accumulation likely results from the EET activity of *S. oneidensis* MR‐1, which facilitates cathodic electron uptake and proton reduction. In contrast, *C. aceticum* in monoculture primarily relies on hydrogen for CO_2_ conversion via WLP, leading to lower hydrogen accumulation.

Collectively, the efficient synergy of the CS co‐culture is reflected in four aspects: metabolic complementarity, optimized electron transfer, the role of hydrogen as an electron donor, and local microenvironment regulation. These synergistic mechanisms collectively enhance electron transfer efficiency and acetate production, highlighting the potential of optimizing biofilm formation and riboflavin‐mediated electron transfer to boost the productivity of MES systems.

Thus, the advantages of the co‐culture consortia stem from the following synergistic mechanisms: First, the increased secretion of riboflavin (CS consortia) facilitated direct electron transfer by binding to outer membrane cytochromes C, thereby enhancing electron transfer rates. Second, *S. oneidensis* MR‐1 efficiently accepted cathodic electrons to generate hydrogen, providing an electron donor for acetogens, while formate functioned as an intermediate metabolite linking CO_2_ conversion to acetate synthesis. Finally, biofilm formation reinforced local electron transfer efficiency, establishing a DIET network within the electroactive biofilm, facilitating interactions between *S. oneidensis* MR‐1and acetogens. These findings were further supported by conductivity and redox potential analyses (Figure S3, Supporting Information). The significantly higher conductivity in the co‐culture consortia indicated more efficient electron transfer pathways, while the negative shift in redox potential confirmed the formation of a reducing microenvironment within the biofilm, favoring CO_2_ conversion.

In conclusion, this study highlights the pivotal role of biofilm structure and the synergistic effects of metabolic cross‐feeding. In systems containing flavin‐secreting strains (e.g., *S. oneidensis* MR‐1) or under conditions of exogenous flavin supplementation, riboflavin significantly enhances MET and boosts EET, thereby improving acetate synthesis efficiency in co‐culture consortia.

### 2.4 Interactions Between *S. oneidensis* MR‐1 and Non‐Electroactive Acetogens in Electron‐Dependent Co‐Cultures

In MES system, *A. woodii* relies on hydrogen as an essential electron donor for acetate synthesis when cultured alone. However, acetate production was still observed below HEP in the AS co‐culture (Figure 1a). This suggests that *A. woodii* may also utilize riboflavin or formate secreted by *S. oneidensis* MR‐1as electron shuttles for electron transfer. Theoretically, the electron transfer and acetate production efficiency in the suspension of the AS co‐culture should surpass that in biofilms due to the freer diffusion of mediators in suspension, which facilitates electron transfer.

To test this hypothesis, we examined the biofilm formation in monoculture of *A. woodii* and AS co‐cultures. *A. woodii* alone exhibited minimal electrode attachment, indicating a potential stress response to electrode electrons (**Figure** **4**a). Although biofilm formation improved in the AS co‐culture, the biofilm remained relatively thin (Figure 4a). Additionally, the capacitance of the *A. woodii* monoculture suspension was high (Figure S4, Supporting Information), with no significant difference from the AS consortia below the HEP, indicating that *A. woodii* primarily grows in suspension. The presence of *S. oneidensis* MR‐1 enhanced its biomass (1.84 ± 0.65 µg cm^−^^3^ in AS vs 1.25 ± 0.17 µg cm^−^^3^ in monoculture, Figure 3i). As shown in Figure 4b, the AS suspension exhibited the strongest riboflavin signature peak at −0.6 V vs SCE, further confirming the occurrence of active electron transfer. Periodic removal of planktonic cells and quantification of acetate production (Figure 4d) revealed that the AS suspension contributed more significantly to acetate production below the HEP (60.11–83.33%) and displayed a similar trend above the HEP (62.60–74.41%). These results suggest that hydrogen and formate generation promote electron utilization and CCE in planktonic cells (Figures 1 and 4c).

Furthermore, the expression levels of functional genes associated with NAD(P)H synthesis, the WLP, and ATPase were significantly upregulated in planktonic cells of the AS consortia (AS‐S) compared to biofilm cells (AS‐B) (Figure 4e). Poised electrodes can drive *S. oneidensis* MR‐1 toward NADH‐dependent catabolism, where electrons entering the quinol pool via the Mtr pathway are reversely converted into NADH.^[^ ^26^, ^27^ ^]^ Such EET‐linked reverse electron transport, also demonstrated in engineered *S. oneidensis*’s MtrCAB systems,^[^ ^28^ ^]^ provides reducing power that supports acetogen‐mediated CO_2_‐to‐acetate conversion in our co‐cultures. This process relies on riboflavin‐mediated electron shuttling and establishes a thermodynamic driving force through the proton gradient.^[^ ^29^, ^30^ ^]^ By flexibly distributing interspecies electrons through formate and riboflavin shuttles, *S. oneidensis* MR‐1 effectively alleviates *A. woodii’*s dependence on hydrogen, enhancing electron transfer efficiency. Compared to a system with exogenous riboflavin alone, the AS co‐culture exhibited higher acetate production efficiency (Figure S6, Supporting Information), underscoring the pivotal regulatory role of *S. oneidensis* MR‐1 in the cathodic biofilm.

As a non‐electrogenic microorganism, *A. woodii* primarily relies on electron distribution in suspension and struggles to metabolize effectively within biofilms. This phenomenon can be attributed to the spatial preference of electron transfer pathways: while DIET is generally confined to the electrode surface, IET expands the electron transfer range through soluble shuttles like riboflavin and formate. Such mechanisms optimize metabolic reactions in the liquid phase, particularly acetate synthesis. During this process, *S. oneidensis* MR‐1 primarily grows as planktonic cells or thin biofilms. When cell‐derived enzymes or cofactors adsorb onto the electrode surface, the overpotential can be significantly reduced, enhancing electrosynthesis efficiency.^[^ ^6^ ^]^ Conversely, if cells are not surface‐attached, planktonic cells must frequently contact the electrode to release stored charges. Through increased riboflavin and multiple electron shuttles like hydrogen and formate (Figure 2f,g), S*. oneidensis* MR‐1establishes efficient electron transfer pathways, supporting the metabolism of non‐electrogenic species like *A. woodii* in suspension and thereby boosting acetate production in the co‐culture consortia. Beyond MES systems, the positive correlation between flavin concentration and the reduction activity of dissolved manganese in marine sediments and pore water further illustrates the universal role of flavins in cross‐species electron transfer.^[^ ^5^ ^]^ Additionally, the dynamic balance between biofilm and planktonic communities is critical for maintaining MES system stability and enhancing efficiency.

Dynamic pH profiles (Figure S7a,b, Supporting Information) revealed distinct microenvironmental adaptations across microbial consortia. As the cathodic potential became lower, OD_600_ values increased in all groups (Figure S7d–f, Supporting Information), suggesting enhanced planktonic growth under electrochemical stimulation. This trend may result from biofilm formation at high potentials or a shift from biofilm‐ to plankton‐dominated growth. However, the weak correlation between OD_600_ and acetate production implies that while growth supports metabolism, it is not the sole driver—possibly reflecting a shift in energy acquisition strategies. Beyond this, spatial organization within the consortia appears to play a key role in maintaining system function.^[^ ^31^, ^32^ ^]^ Interactions between biofilm‐forming and planktonic populations may promote ecological niche partitioning,^[^ ^33^ ^]^ allowing free‐living acetogens and electroactive biofilm‐formers to coexist and respond flexibly to cathodic conditions. Supporting this view, we observed a sharp decline in acetate levels following planktonic cell removal (Figure S7f–i, Supporting Information). Interestingly, biofilm cells tended to detach and re‐enter suspension shortly after removal events (Figure S7c, Supporting Information), a phenomenon consistent with previous reports showing that periodic removal of planktonic cells can stimulate biofilm growth on cathodes.^[^ ^8^ ^]^

At the metabolic level, the CS co‐culture demonstrated that *C. aceticum* consumes protons during CO_2_ fixation, generating a proton gradient to support ATP synthesis. In contrast, the AS system features a more alkaline microenvironment, where *A. woodii*, relying on Na⁺‐dependent ATPases,^[^ ^7^ ^]^ exhibits better adaptability. Despite a weakened proton motive force (PMF), *S. oneidensis* likely sustains energy metabolism through riboflavin‐mediated EET. Consistently, pronounced current peaks were observed in the AS system (Figure 2d; Figure S9, Supporting Information), possibly reflecting accumulated electron shuttles or enhanced electron transfer efficiency. These results underscore the importance of regulating the spatial distribution and migratory dynamics of microbial consortia to ensure the stability and resilience of MES systems, highlighting the essential role of planktonic communities in sustained electrosynthesis.

### 2.5 *S. oneidensis* MR‐1 as an Ecosystem Engineer Regulating the Electron Flux Between Different Functional Bacteria and Position

In natural ecosystems, over 98% of CO_2_ is assimilated via CO‐catalyzed carboxylation,^[^ ^34^ ^]^ yet environmental constraints limit its efficiency and directed utilization. MES provides a solution by enabling precise electrochemical control, optimizing electron donor utilization, and stabilizing CO_2_ conversion for industrial applications.

**Figure 5:**
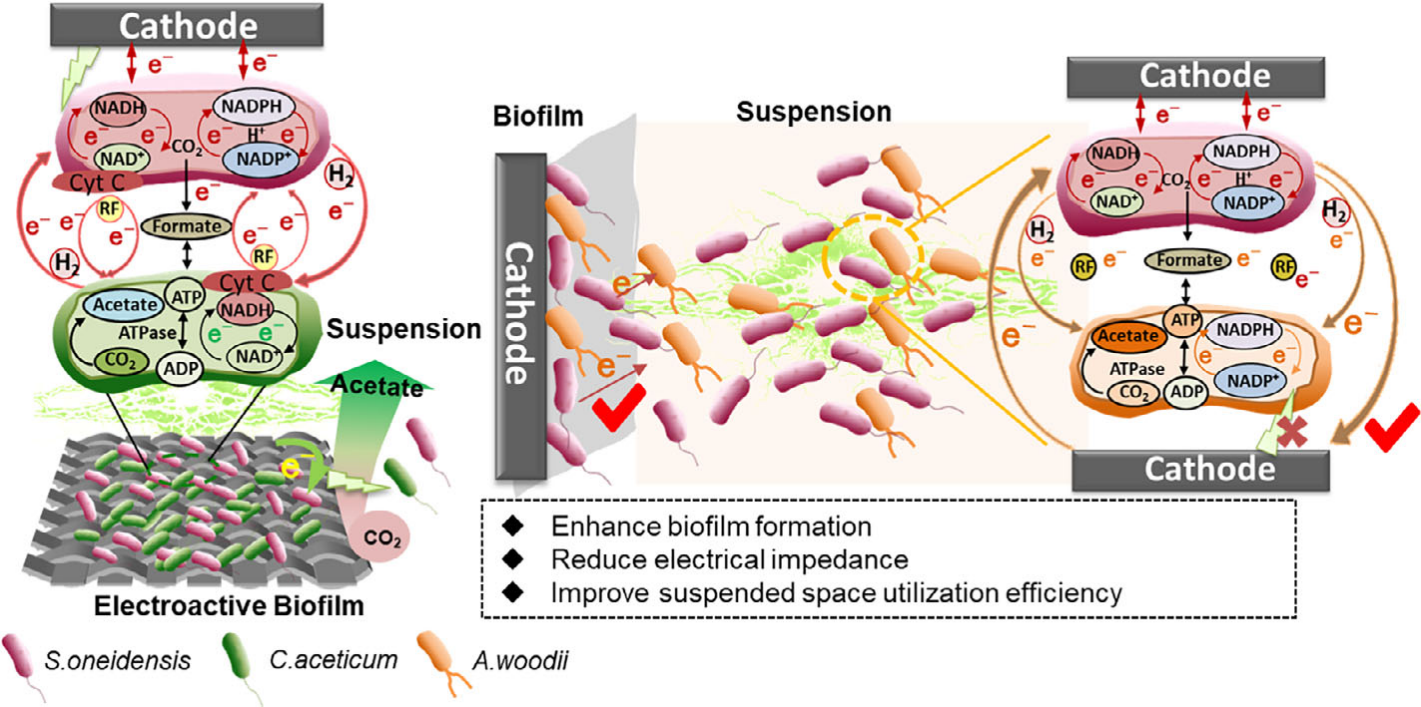
The electronic interaction between *S.oneidensis* MR‐1 and electroactive *C.aceticum* and non‐electroactive *A.woodii* in biofilms and suspension spaces produces acetate.

In MES co‐culture, *S. oneidensis* MR‐1 functioned as an “ecosystem engineer,” dynamically regulating electron transfer. By generating metabolic intermediates (H_2_, formate), it facilitated flexible electron distribution and community‐level metabolic specialization (**Figure** **5**). Under high overpotential (−0.80 V vs SCE), *S. oneidensis* MR‐1 produced H_2_ as a key MET mediator, significantly enhancing metabolic activity (Figure S1, Supporting Information). Additionally, riboflavin acted as an electron shuttle, indirectly boosting proton pump activity, while the accelerated circulation of formate and acetate sustained PMF and NADH/NADPH regeneration for CO_2_ fixation.^[^ ^6^, ^25^, ^35^ ^]^ This process reflects a highly dynamic interplay of electron flow, metabolic division of labor, and regulatory flexibility, ensuring efficient product synthesis and system stability.

This electron transfer‐driven coordination resulted in a distinct spatial division within the consortia: *C. aceticum* engaged in cytochrome‐based DIET at the biofilm–electrode interface, whereas the non‐electroactive *A. woodii* depended on mediator‐driven MET in the planktonic phase. These complementary strategies jointly shaped electron flux and product distribution, with MET extending acetate synthesis beyond the biofilm. Integrating both biofilm‐ and suspension‐oriented populations, such as in a three‐membered consortium of *S. oneidensis* MR‐1, *C. aceticum*, and *A. woodii*—could thus optimize electron allocation and spatially coordinated production in MES systems.

In methanogenic environments, *Geobacter* spp.—particularly *G. sulfurreducens*—serve as canonical DIET performers, transferring electrons to methanogens such as *Methanosaeta* and *Methanosarcina* via conductive *pili* (microbial nanowires) and multiheme cytochromes like OmcS.^[^ ^36^, ^37^, ^38^ ^]^ These *pili‐*based connections enable direct electron exchange but require intimate contact and strictly anaerobic conditions, limiting their flexibility in dynamic MES systems. In contrast, *S. oneidensis* MR‐1 integrates cytochrome‐based DIET with flavin‐, H_2_‐, and formate‐driven MET, enabling spatially flexible and efficient interspecies electron transfer. While molecular mechanisms differ among exoelectrogens, the conceptual framework established here—linking electron transfer modes to spatial product formation—offers generalizable insight for engineering structured and efficient MES consortia.

## 3 Conclusion

This study advances microbial electrosynthesis by constructing defined consortia to enhance CO_2_ conversion. Pairing *Shewanella oneidensis* MR‐1 with *Clostridium aceticum* or *Acetobacterium woodii* revealed distinct interspecies electron transfer mechanisms, boosting acetate production substantially and achieving high carbon conversion efficiencies over 84%. *S. oneidensis* MR‐1 acts as a key ecosystem engineer, managing electron flow through biofilm‐localized DIET or H_2_/formate‐mediated pathways. These insights lay the groundwork for rational design of MES systems, contributing to innovative carbon fixation strategies.

## 4 Experimental Section

### Bacterial Inoculum and Culture Medium

The bacterial inoculum consist of: A‐*A. woodii* (DSM 1030), S‐*S. oneidensis* MR‐1 (MCCC ATCC 700550), and C‐*C. aceticum* (DSM 1496). The culture medium for these bacteria is an optimized DSMZ‐Medium135 base medium (1L) containing 0.25 g NH_4_Cl, 0.31 g KH_2_PO_4_, 0.33 g K_2_HPO_4_, 0.2 g MgSO_4_·7H_2_O, 1 mL trace elements, 1 mL vitamins (DSMZ‐Medium 141), 0.1 g yeast extract, 3.5 g NaHCO_3_, 0.25 g L‐cysteine, 0.25 g Na_2_S·9H_2_O, and 0.5 mL 1% methylene blue. The formula for trace elements and vitamin solutions is detailed in the supplementary materials.

### Reactor Configuration, Startup, and Operation

The reactor consists of an H‐type dual‐chamber design, where a proton exchange membrane (Nafion 117) separates the anode and cathode compartments (Figure S8, Supporting Information). Each chamber has an effective volume of 250 mL. Carbon cloth (3.5 cm × 5 cm × 0.036 cm, Taiwan Carbon Energy W02011) serves as both the anode and cathode electrodes. The two chambers are electrically connected via a 10 Ω resistor, with titanium wires securing the electrodes.^[^ ^39^ ^]^ A DC power supply (3645A, Array Electronics) provides voltage to the system, while real‐time data acquisition is carried out using a multi‐channel recorder. All electrode potentials in this study are referenced to a saturated calomel electrode (SCE, Leici 217; +0.230 V vs SHE at 40 °C), unless otherwise stated. All experiments in this study were conducted in triplicate independent reactors (*n* = 3) for each experimental condition unless otherwise specified.

In the reactor startup phase, *S. oneidensis* MR‐1 was co‐cultured with *A. woodii* and *C. aceticum* in various combinations (denoted as AS and CS), with separate control groups where only *A. woodii* or *C. aceticum* was added. The cathode chambers were based on the culture medium, with 5 mL of each bacterial culture at the logarithmic growth phase (OD_600_ = 0.5) inoculated into the system. Samples were taken every 2d to measure OD_600_, pH, acid production, and headspace gas composition, and each cycle lasted 7d. The reactor was operated for three sequential cycles. After each cycle, fresh medium was added to maintain the initial volume. The anolyte was a phosphate buffer solution (50 mm, pH 7.0) containing 20 g L^−1^ potassium ferrocyanide. The applied voltages were set to 0.88 and 0.54 V, respectively. The reaction was conducted in a thermostatic chamber (37 °C) with a headspace atmosphere of N_2_/CO_2_ (80:20). In open‐circuit experiments, the working electrode was disconnected. When the headspace atmosphere was N_2_/CO_2_, single‐acetogens strain cultures failed to synthesize acetate efficiently. Therefore, H_2_/CO_2_ (80:20) was specifically added as the headspace gas for open‐circuit experiments to provide the minimal electron donor required for acetate formation. All other conditions were consistent with the electrochemical operation. Under applied voltage of 0.54 V, the role of exogenous riboflavin in mediating IET between *S. oneidensis* MR‐1and the acetogen was assessed. Riboflavin concentration in the MES system was measured to be at micromolar levels, and therefore exogenous riboflavin was added at a concentration of 10 µm.

### Quantification of Biofilm and Planktonic Contributions to MES Cathodic Synthesis

To quantify the relative contributions of biofilm‐and planktonic‐associated microorganisms to acetate production in MES, the experimental group was operated with daily removal of planktonic liquid, followed by centrifugation and filtration (0.22 µm) to eliminate suspended cells, after which the cell‐free supernatant was reintroduced into the reactor. The control group was operated under identical conditions without planktonic removal.

A normalization framework was applied to calculate the daily contribution rates. The control system (without planktonic removal) served as the denominator, while the experimental system reflected biofilm‐derived acetate production; the difference between the two conditions indicated the planktonic fraction. The daily instantaneous contribution rate was calculated as:

(1) $$Daily\;instantaneous\;contribution\;rate\;\left(\%\right)=\frac{C_{Acetate,\;fraction\;,\;day}}{C_{Acetate,\;control\;,\;day}}\times 100\%$$

where *C*_*Acetate*, *fraction*,  *day*_ is the acetate produced by the tested fraction (biofilm or planktonic) on a given day, and *C*_*Acetate*, *control*,  *day*_ is the total acetate yield of the control system on the same day. Because this calculation is based on instantaneous daily yields rather than cumulative totals, values may not strictly sum to 100% and can occasionally exceed 100% due to transient washout or compensation effects.

### Analyses and Calculations

The gas components (H_2_, N_2_, and CO_2_) were analyzed using a gas chromatograph (GC‐9790 II, FULI WORKS, China). Carboxylic acids and alcohols were detected using another gas chromatograph (GC‐2010, SHIMADZU, Japan). The pH of the samples was measured with a pH meter (INESA Scientific Instrument Co., LTD, China). The optical density of the samples at 600 nm was measured using a UV spectrophotometer (UV‐1600, MAPADA INSTRUMENTS, China). The cathode potential and current were monitored online by a data collector (Keithley‐2700/E, USA).^[^ ^40^ ^]^ The electrochemical characteristics of the biocathode were characterized by cyclic voltammetry (CV) and electrochemical impedance spectroscopy (EIS) using an electrochemical workstation (CHI600E, CHINSTRUMENTS, China), with parameters referenced from a previous study.^[^ ^41^ ^]^ The calculation of carbon conversion efficiency (CCE) and electron transfer efficiency (ETE) refers to the calculation formulas of previous studies.^[^ ^42^ ^]^ Statistical difference analyses were performed by IBM SPSS Statistics 25 software.

After stabilizing the system for 2 h under open‐circuit conditions, bio‐cathode EIS testing was conducted by applying a small‐amplitude sinusoidal AC signal and recording the response. Parameters such as solution resistance (R_1_), biofilm resistance (R_2_), charge transfer resistance (R_3_), nonideal capacitance (C_1_), and double‐layer capacitance (CPE_1_, F) were determined through equivalent circuit fitting. The DPV test potential ranged from −0.7 to 0.4 V vs SCE, while the Tafel test was conducted within the potential range of −0.6 V to 0.6 V/1.2 V vs SCE. Electrochemical characterizations were carried out using a three‐electrode system. The working electrode potentials were 0.61 V for the electron acceptance capacity (EAC) and −0.49 V for the electron donation capacity (EDC). EDC and EAC values were determined by integrating the oxidative and reductive current from chronoamperometric i–t curves.^[^ ^23^ ^]^

Details on EPS extraction, cytochrome *c* detection, and riboflavin quantification are provided in the Supporting Information. The protein content was quantified using a BCA kit, soluble total sugars were measured via the phenol‐sulfuric acid method, and soluble proteins were quantified using the Lowry‐Folin method, with bovine serum albumin as the standard.^[^ ^33^ ^]^ Quantification and standardization of biomass in suspensions and on electrode biofilms are detailed in the Supporting Information. Afterward, DNA was extracted from biofilms on the bio‐cathode and their corresponding planktonic microbes. 16S rRNA sequencing was performed, and real‐time quantitative PCR was used to analyze the gene encoding 10‐formyl tetrahydrofolate synthetase (*fhs*) and total microbial biomass.^[^ ^43^ ^]^ Microbial community analysis was carried out using the Illumina MiSeq platform, with bacterial 16S rRNA sequencing primers 338F and 806R.^[^ ^44^ ^]^

### Statistical Analysis

Data analysis of key enzyme abundance first normalized raw read counts to eliminate technical variation between samples, then calculated the log2 fold change (FC) of normalized data to quantify relative changes in gene abundance across different samples. Comparisons between different groups were performed using one‐way analysis of variance (ANOVA), followed by Tukey's multiple comparison test or Kruskal–Wallis test with Dunn's multiple comparison test to identify significant differences. All statistical significance tests in the experiments were two‐tailed. Results were considered statistically significant when *p*‐values and FDR q‐values were <0.05. Data are presented as mean ± standard deviation, with sample sizes (*n* = 3, replicates) for each statistical analysis. Software used for statistical analysis included SPSS, Origin, and GraphPad Prism. Further details are provided in the Supplementary Information.

## Conflict of Interest

The authors declare no conflict of interest.

## Supporting information



Supporting Information

## Data Availability

The data that support the findings of this study are available from the corresponding author upon reasonable request.
