# Supplementary material for: Defined Electrosynthetic Microbial Consortia Reveal Electron Transfer Modes Governing Acetate Production
Source: Adv Sci (Weinh). 2025 Nov 14;13(6):e13340. doi: 10.1002/advs.202513340 (PMC12866739; doi:10.1002/advs.202513340)
Supplement: Supplementary file 1 — Supporting Information [file ADVS-13-e13340-s001.docx]

**Appendix. A Supplementary materials**

Jing Zhang^a^, He Liu^a,b*^, Qihao Cao^a^, Chao Zhang^a^, Min Zhang^a^, Minhua Cui^a^,

Yan Zhang^a^, Bo Fu^a^, Hongbo Liu^a,b^

^a^*School of Environment and Ecology, Jiangnan University, Wuxi 214122, China*

^b^*Jiangsu Collaborative Innovation Center of Water Treatment Technology and Material, Suzhou 215011, China*

*Corresponding author at: School of Environment and Ecology, Jiangnan University, Wuxi 214122, China.

E-mail address: liuhe@jiangnan.edu.cn (He. Liu).

1. **Materials and Methods**

**1. Materials and Culture Medium**

The trace elements solution (1L) is prepared as follows: 1.50 g tris(hydroxymethyl) aminomethane, 0.10 g CaCl₂·2H₂O, 3.00 g MgSO₄·7 H₂O, 0.01 g CuSO₄·5H₂O, 0.50 g MnSO₄·H₂O, 0.01 g H₃BO₃, 1.00 g NaCl, 0.03 g NiCl₂·6H₂O, 0.10 g FeSO₄·7H₂O, 0.40 mg Na₂WO₄·2H₂O, 0.18 g CoSO₄·7H₂O, 0.18 g ZnSO₄·7H₂O, 0.02 g KAl(SO₄)₂·12H₂O, 0.01 g Na₂MoO₄·2H₂O, and 0.30 mg Na₂SeO₃·5H₂O. First, dissolve tris(hydroxymethyl)aminomethane and adjust the pH to 6.5 with KOH solution. Then, sequentially dissolve the other salts. Once dissolved, adjust the final pH to 7.0 with KOH solution. The vitamins solution (1L) includes: 2 mg biotin, 10 mg pyridoxine hydrochloride, 2 mg folic acid, 5 mg p-aminobenzoic acid, 5 mg thiamine hydrochloride·2 H₂O, 5 mg D-calcium pantothenate, 5 mg riboflavin, 0.10 mg vitamin B12, 5 mg niacin, and 5 mg α-lipoic acid.

Before use, the carbon cloth undergoes pretreatment, including a 12 h soak in acetone, thorough rinsing with deionized water, and immersion in a 30% hydrogen peroxide solution. It is then heated at 80°C in a water bath for 2 h, followed by multiple washes with ethanol and deionized water before being dried at 105°C. This treatment enhances hydrophilicity and promotes microbial adhesion. The proton exchange membrane is pretreated with 5% hydrogen peroxide and 5% sulfuric acid solutions and stored in deionized water.

**2. Analyses and calculations**

CCE = $\frac{\text{2C}_{\text{Acetate}}}{\text{C}_{\text{NaHCO3}}}\text{×100\% }$ (1-1)

Where C_Acetate_ (mol/L) represents concentration of acetate produced, C_NaHCO3_ (mol/L) is concentration of NaHCO_3_ added.

ETE =$\frac{\text{FCVK}}{\int_{\text{t=0}}^{\text{t}} \text{Idt}}\text{×100\%}$ (1-2)

Where F is Faraday's constant, 96485 C/mol, C (mol/L) represents concentration of acetate produced, V (L) is volume of catholyte, and K is equivalent electrons required to generate each mole of product (8 mol eq/mol for acetate), I (A) represents the reaction current, t (s) represents reaction time.

After obtaining the CV curve, the sample’s specific capacitance (Cₐ, mF/cm²) can be calculated using Formula 1-3.

$Ca=\frac{\int_{v1}^{v2} I(V)dv}{2Sv(V_{2}-V_{1})}$ (1-3)

Where S (cm²) represents the projected area of the bio-cathode, v (V/s) is the scan rate, V₁ (V) is the starting potential, and V₂ (V) is the ending potential.

Extracellular polymeric substances (EPS) are categorized into soluble EPS (S-EPS), loosely bound EPS (LB-EPS), and tightly bound EPS (TB-EPS). The separation of EPS was performed using an improved heat extraction method. Bio-cathode carbon cloth samples were first collected, centrifuged (4000 rpm, 5 min, 4°C), and filtered through a 0.45 μm membrane to obtain S-EPS. Subsequently, a 0.9 % NaCl solution was added, vortexed for 2 min, centrifuged, and the supernatant discarded. A 70°C NaCl solution was then added, followed by centrifugation for 15 min to obtain LB-EPS. Finally, a 0.05% NaCl solution was added, and the mixture was heated in a 60°C water bath for 30 min, centrifuged for 30 min, and filtered to obtain TB-EPS.

The 3D excitation-emission matrix (3D-EEM) spectra of the EPS samples were analyzed using a Horiba JY Aqualog fluorescence spectrometer. The emission and excitation wavelength ranges were both 250~500 nm, with a step size of 4.48 nm, a resolution of 5 nm, and a scan rate of 12000 nm/min.

**Biomass quantification and normalization:** The biomass of electrode-attached and planktonic microorganisms was determined separately and standardized to a uniform volumetric unit (μg cm⁻³) to enable direct comparison. For biofilm samples, the protein content was first measured as μg cm⁻² based on the electrode surface area and then converted to volumetric biomass (μg cm⁻³) using the measured average biofilm thickness of each electrode. For suspension samples, the protein concentration in the liquid phase was directly measured as μg mL⁻¹ and expressed as μg cm⁻³ (1 μg mL⁻¹ = 1 μg cm⁻³). All biomass data are reported as mean ± standard deviation (n = 3).

**Cytochrome *c* Detection:** The bacterial cells were first centrifuged at 8000 rpm for 5 min and washed 1~2 times with PBS. The cells were then resuspended in 1 mL of SL solution (containing 20% sucrose, lysozyme, and Tris buffer) and incubated at 35°C for 1 h on a shaking incubator. After centrifugation at 8000 rpm for 5 min, the pellet was resuspended in Tris-Mg^2+^ solution (containing Tris and MgCl_2_) and incubated at 35°C for 20 min. The supernatant was collected by centrifugation, yielding a mixture of bacterial outer membrane and periplasmic proteins. The sample was subjected to ultrasonic disruption for 30 min, filtered, and analyzed using a UV spectrophotometer with a wavelength range of 350~600 nm.

Riboflavin content was quantified by high-performance liquid chromatography (HPLC) with a C-18 column, using a mobile phase of 0.1% formate aqueous solution and acetonitrile, at a flow rate of 0.3 mL/min with gradient elution, and a 10 μL injection. Riboflavin concentrations were determined by comparing the retention peak areas with those of a standard. The reaction supernatant was then centrifuged, filtered, and used for fluorescence scanning with an excitation wavelength of 380 nm and an emission range of 470~650 nm.

**
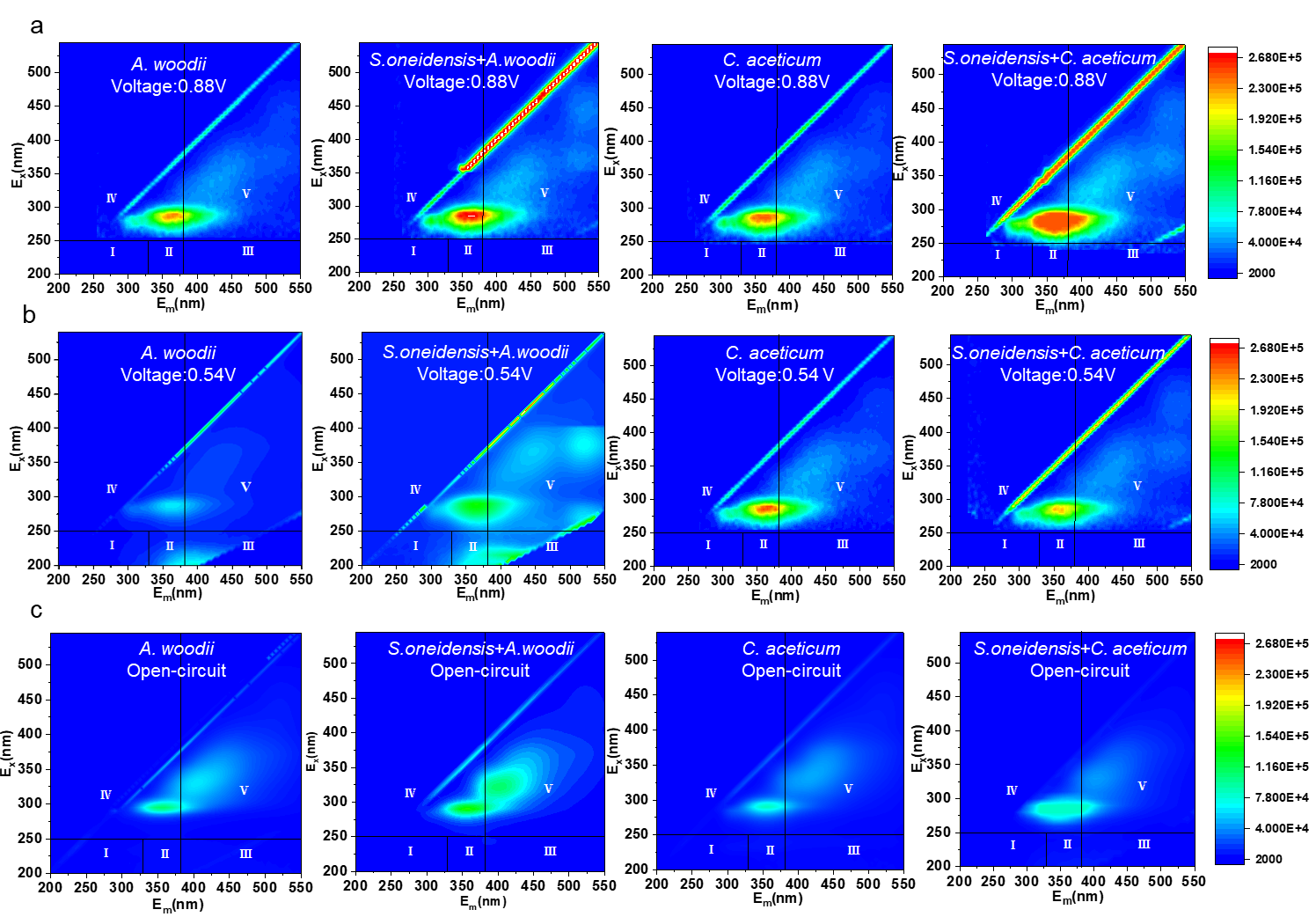
**

Fig. S1. 3D fluorescence maps of each pure and mixed culture system (a) above the hydrogen precipitation potential and (b) below the hydrogen precipitation potential and (c) without the potential

*
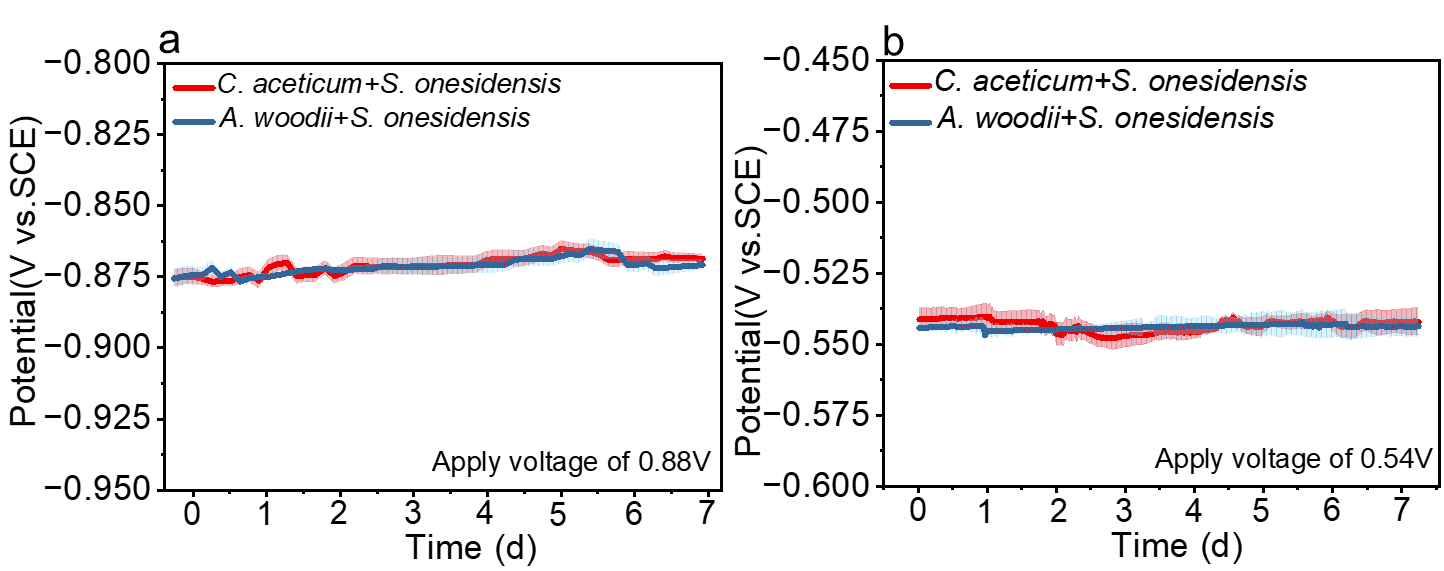
*

Fig. S2. Timing curves of cathode potential (vs. SCE) measured during operation with applied battery voltages of (a) 0.88 V and (b) 0.54 V. The shaded area represents experimental error(n=3).


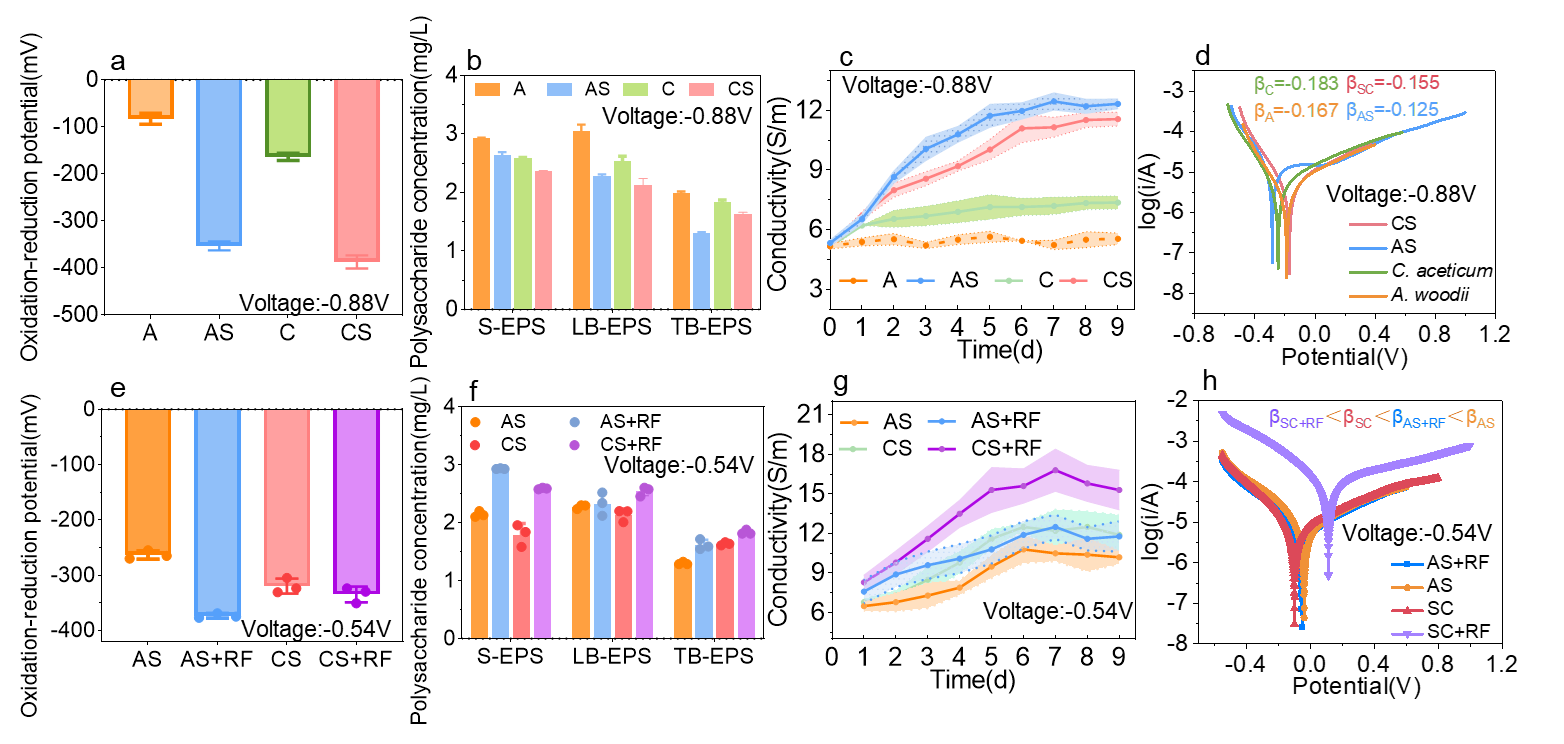


Fig. S3. (a, e) Redox potentials with applied battery voltages of 0.88 V and 0.54 V MES; (b, f) Polysaccharide composition in each EPS layer at the cathode (S: soluble EPS; LB-EPS: loosely bound EPS); (c, g) Conductivity changes of aggregate reactions above and below the hydrogen evolution potential; (d, h) Tafel curves of the cathode at corresponding voltages.


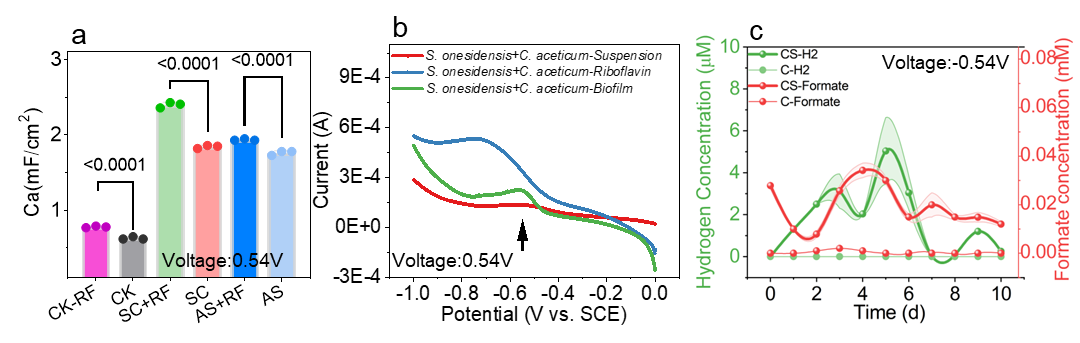


Fig. S4. (a) unit capacitance; (b) LSV curves of the CS co-culture biofilm and suspension; (c) changes in hydrogen and formate in the CS consortia.


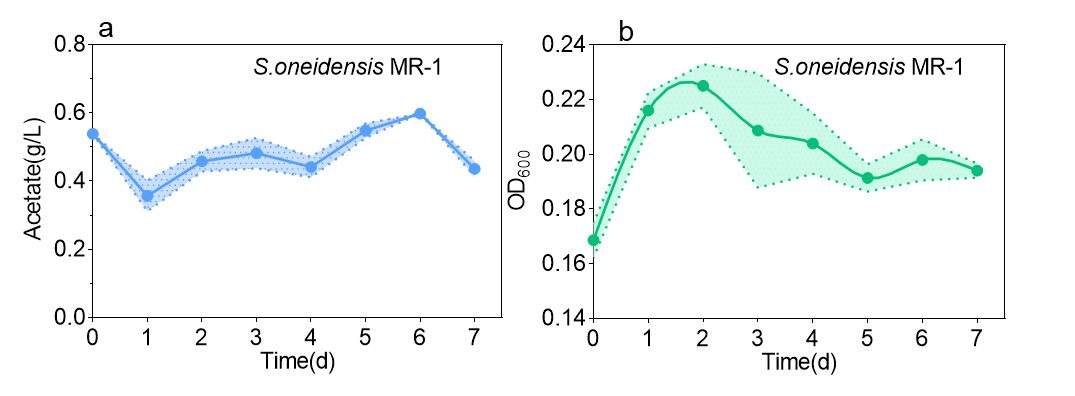


Fig. S5. (a) growth of *S. oneidensis* in a culture medium with acetate as the carbon source; (b) utilization of acetate


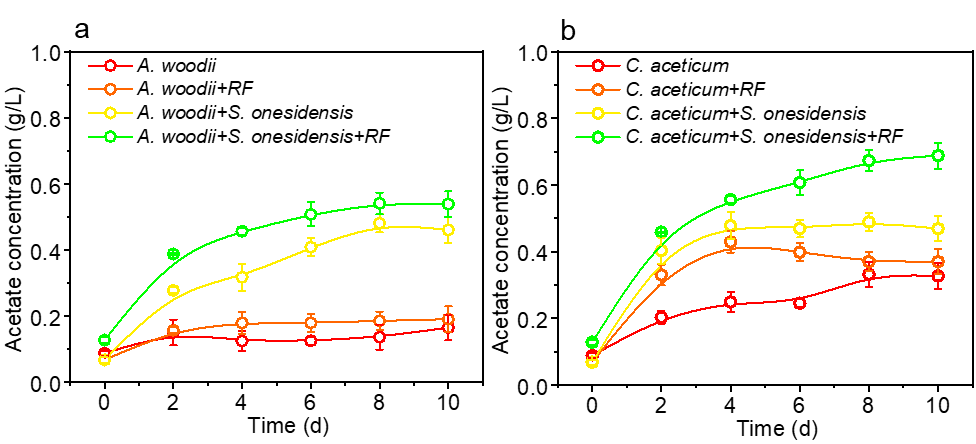


Fig. S6. Acetate titer of (a) AS and (b) CS consortia with exogenous addition of RF with applied battery voltages of 0.54V


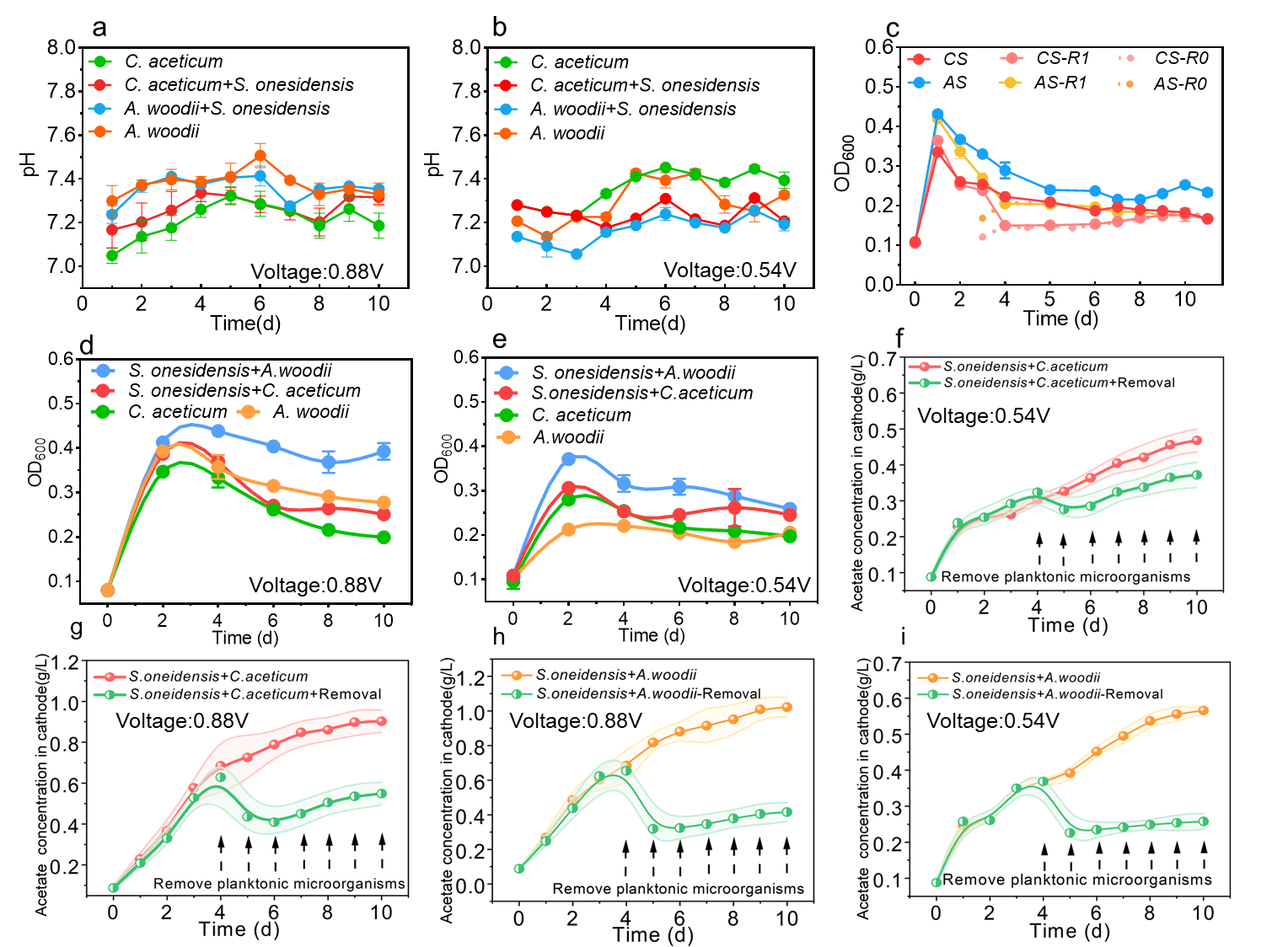


Fig. S7. pH changes of cathode suspensions in each MES system under applied voltage of (a) 0.88V (b) 0.54V; (c) OD_600_ changes for the consortia with removal of planktonic microorganisms under applied voltage of 0.54V, R0 represents the instantaneous measurement after removal of planktonic microorganisms on the same day; OD_600_ changes for the consortia e) under applied voltage of 0.54V; acetate production for CS (f) under applied voltage of 0.54 V; (g) under applied voltage of 0.88 V, with planktonic removal; acetate production for (h) AS under applied voltage of 0.54 V; and (i) under applied voltage of 0.88 V.


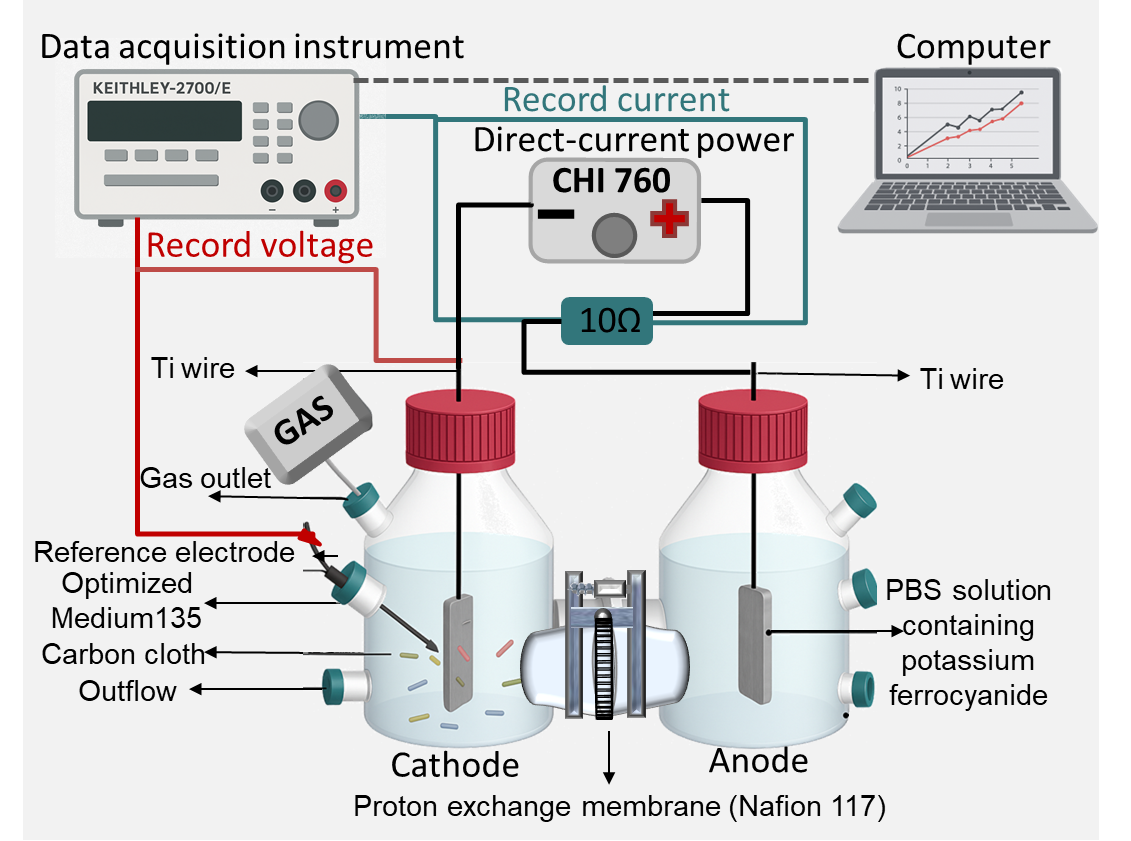


Fig. S8. (a) Reactor model and circuit connection diagram


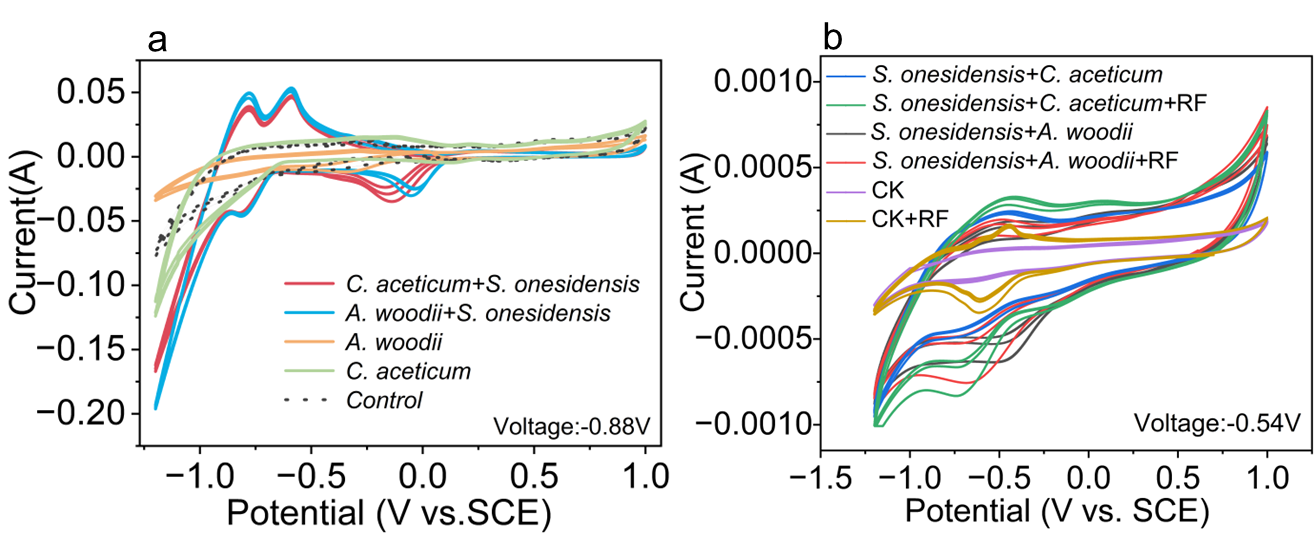


Fig. S9. (a) The CV curve shows three sets of repetitions at voltages of 0.88V and 0.54V

Table S1. Benchmark Comparison of MES Performance Parameters with Representative Studies

| Acetogen strain/consortium | Operating mode / headspace gas | Cathode potential (vs. SHE) | Maximum acetate titer (g/L) | ETE/CE for acetate (%)/ | Acetate’s CCE (%) | Ref |
| --- | --- | --- | --- | --- | --- | --- |
| *Sporomusa sphaeroides* | Initially, the ratio was N₂:CO₂:H₂ = 83:10:7; subsequently, N₂:CO₂ = 80:20 | -0.4 V | 0.00319 |  | 27± 1.5 | ^[1]^ |
| *Sporomusa silvacetica* | Same as above | -0.4 V | 0.00236 |  | 39 ±1.5 | ^[1]^ |
| Mixed culture | Constant pressure | -1.359 V | 0.599 | 13.20 | 44.31±0.24 | ^[2]^ |
| Mixed culture | Constant Pressure/H₂ Partial Pressure Regulation | -0.759V | 1.12 | 71.06±2.71 | 69.75±0.92 | ^[2]^ |
| *Acetobacterium* (dominant) | Adjust pH to 5.4, Continuous flow; 2 mM fructose addition  CO_2_:N_2_=90:10 | -0.803 V | — | 83.2±10.5 | 81 | ^[3]^ |
| Acetogenic microbiome | Continuous flow/Constant flow | -1.1 ~ -1.3V | — | 11.2±0.8 | 35 | ^[4]^ |
| *Acetobacterium* (96.5%) | Continuous flow; salinity of 35 g/L  N_2_:CO_2_=90：10 | -1.003 V | — | 55.1 | — | ^[5]^ |
| *Sporomusa ovata* | H₂:CO₂ = 80:20 startup; 100% CO₂ continuous bubbling | -0.680 ± 0.025 V | 0.44 ± 0.22 | — | 2.47 ± 0.12 | ^[6]^ |
| *Thermoanaerobacter kivui* | Intermittent constant flow. H_2_: CO_2_=80:20  0.7 bar overpressure;65℃ | — | 3.54 ~ 5.31 | — | 96 | ^[7]^ |
| *Shewanella oneidensis* MR-1 | Ammonium regulation | -0.4 ~ -0.8 V | 0.309 ± 0.023 | 72 ± 11（formate and acetate） | 63 ± 10 | ^[8]^ |
| *Mixed sludge + MR-1 + FeS* | Constant pressure | −0.759V | 1.35 | 75.78 | 63.10 | ^[9]^ |
| *Shewanella oneidensis MR-1+ Acetobacterium woodii* | 35℃; NaHCO_3_;  N₂:CO₂ = 80:20 | -0.65± 0.025V | 1.16 ± 0.01 | 75.58 ± 1.61 | 93.12 ± 0.56 | ^this study^ |
| *Shewanella oneidensis MR-1+ Clostridium aceticum* | Same as above | -0.65± 0.025V | 1.05 ± 0.01 | 84.40 ± 2.63 | 84.03 ± 1.01 | ^this study^ |

Note: Studies were selected based on the following criteria—(i) CO_2_ as sole carbon source; (ii) acetate as main product; (iii) clearly reported cathodic potentials (vs. SCE/Ag-AgCl/SHE); (iv) defined electroactive or acetogenic strains under MES operation; and (v) unmodified electrode materials. These criteria ensure comparability in electrochemical configuration rather than absolute titer performance.

Table S2. Component parameters of the equivalent circuit in the EIS diagram of electrochemical impedance spectra under applied voltage of 0.88V

|  | **R1(Ω)** | **Error（%）** | **R2（Ω）** | **Error（%）** | **R3（Ω）** | **Error（%）** | **C1（F）** | **Error（%）** | **CPE1（F）** | **Error（%）** |
| --- | --- | --- | --- | --- | --- | --- | --- | --- | --- | --- |
| **AS** | 5.19±1.96 | 4.00 | 17.92± 1.03 | 5.51 | 22.74± 1.03 | 1.03 | 5.61E-08 | 7.78 | 0.73 | 6.88 |
| **CS** | 4.30±0.56 | 3.31 | 8.71± 0.89 | 0.51 | 12.36± 1.83 | 13.76 | 3.88E-08 | 1.01 | 0.85 | 2.60 |
| **C** | 5.25±1.13 | 1.01 | 16.92± 1.68 | 7.54 | 21.34± 3.06 | 14.82 | 4.39E-08 | 11.39 | 0.77 | 3.63 |
| **A** | 6.27±0.63 | 3.00 | 19.83± 2.25 | 2.63 | 36.56± 5.08 | 6.82 | 3.31E-08 | 5.96 | 0.75 | 3.68 |
| **CK** | 10.76±1.19 | 9.23 | 20.9± 1.89 | 3.69 | 30.4± 4.02 | 11.2 | 2.01E-08 | 6.83 | 0.70 | 2.63 |

Table S3. Headspace gas composition in MES reactors after 7 days of operation. Values represent mean ± SD (n = 3).

| **Voltage/Condition** | **Culture type** | **H₂ (μmol/L)** | **N₂ (%)** | **CO₂ (%)** |
| --- | --- | --- | --- | --- |
| **0.8 V**  N_2_: CO_2_=80:20 | AS consortium | 8.05 ± 1.96 | 78.36 ± 0.94 | 1.56 ± 0.85 |
|  | CS consortium | 5.18 ± 9.69 | 82.05 ± 0.89 | 2.08 ± 0.82 |
|  | *A. woodii* monoculture | 0.04 ± 0.01 | 87.12 ± 0.81 | 9.9 ± 0.51 |
|  | *C. aceticum* monoculture | 0.08 ± 0.01 | 89.85 ± 0.79 | 6.77 ± 0.47 |
| **0.5 V**  N_2_: CO_2_=80:20 | AS consortium | 0.04 ± 0.01 | 95.12 ± 1.05 | 1.93 ± 0.87 |
|  | CS consortium | 0.04 ± 0.01 | 90.86 ± 0.98 | 2.13 ± 0.92 |
|  | *A. woodii* monoculture | 0 | 80.12 ± 0.31 | 19.20 ± 0.51 |
|  | *C. aceticum* monoculture | 0 | 91.66 ± 0.39 | 8.41 ± 0.47 |
| **Open-circuit**  N_2_: CO_2_=80:20 | AS consortium | 0 | 81.01 ± 0.82 | 18.04 ± 0.49 |
|  | CS consortium | 0 | 80.28 ± 0.77 | 18.89 ± 0.45 |
|  | *A. woodii* monoculture | 0 | 80.12 ± 0.81 | 19.95 ± 0.63 |
|  | *C. aceticum* monoculture | 0 | 79.85 ± 0.69 | 19.41 ± 0.28 |

Table S4. Diagnostic markers distinguishing DIET and MET: expected trends and experimental observations in this study

| Marker (measurement) | Expected in DIET | Expected in MET | Observation Position |
| --- | --- | --- | --- |
| Cytochrome-associated features: DPV/CV peaks near −0.244 V vs SCE; UV–Vis c-type bands (410/530/565 nm) | ↑Peak intensity; ↑cytochrome absorbance (biofilm-localized) | No systematic increase | CS: stronger −0.244 V features & c-type bands (UV–Vis in Fig. 2a; Fig. 2d) |
| Riboflavin features: DPV/LSV window ~ −0.6 to −0.25 V vs SCE; 520 nm fluorescence | Secondary/limited (often biofilm-bound) | ↑Peak intensity & fluorescence (often suspension-enriched) | AS: riboflavin peak prominent in suspension (Fig. 3b, 3c); CS: biofilm-associated RF signal (Fig. 2d, 3a, S4b) |
| EIS: R₂ (biofilm) & R₃ (charge-transfer) | R₂↓, R₃↓ (biofilm conductivity/DIET) | R₁↓, R₃↓ (mediator-enhanced solution) | CS: R₂/R₃ ↓ (Fig. 2i, 3g; Table S2); AS: R₁/R₃↓ (Fig. 2i; Table S2) |
| Biofilm capacitance (CV-derived) | ↑electron storage in conductive biofilm | Modest/variable | ↑in CS (Fig. 2d, 2e, 3c, S4a); moderate in AS |
| EAC/EDC | ↑with DIET-enriched biofilms | with mediator-rich suspensions | ↑in co-cultures; CS aligns with biofilm DIET; AS with MET (Fig. 2c, d) |
| Metabolites:  H₂, formate | Background/auxiliary | mediator-driven IET | AS & CS show H₂/formate↑ vs monocultures; stronger in AS (Fig. 2g, 2j, S4c, 4c) |
| Biomass partitioning / *fhs* copies | Biofilm ↑ | Suspension ↑ | CS: biofilm biomass & *fhs*↑ (Fig. 3e, h); AS: suspension biomass↑ (Fig. 3e) |

**References**

[1] Nevin K P, Hensley S A, Franks A E, et al. Electrosynthesis of Organic Compounds from Carbon Dioxide Is Catalyzed by a Diversity of Acetogenic Microorganisms[J]. Applied and Environmental Microbiology, 2011,77(9):2882-2886.

[2] Zhang C, Cao Q, Zhang J, et al. Regulatory Mechanisms of Electron Supply Modes for Acetate Production in Microbial Electrosynthesis System[J]. ACS Sustainable Chemistry & Engineering, 2025,13(11):4406-4417.

[3] Izadi P, Fontmorin J, Lim S S, et al. Enhanced bio-production from CO_2_ by microbial electrosynthesis (MES) with continuous operational mode[J]. Faraday Discussions, 2021,230(0):344-359.

[4] LaBelle E V, May H D. Energy efficiency and productivity enhancement of microbial electrosynthesis of acetate[J]. Frontiers in microbiology, 2017,8:756.

[5] Zhang X, Arbour T, Zhang D, et al. Microbial electrosynthesis of acetate from CO2 under hypersaline conditions[J]. Environmental Science and Ecotechnology, 2023,13:100211.

[6] Li X, Angelidaki I, Zhang Y. Salinity-gradient energy driven microbial electrosynthesis of value-added chemicals from CO_2_ reduction[J]. Water Research, 2018,142:396-404.

[7] Deutzmann J S, Kracke F, Gu W, et al. Microbial Electrosynthesis of Acetate Powered by Intermittent Electricity[J]. Environmental Science & Technology, 2022,56(22):16073-16081.

[8] Zhang K, Chen J, Zou L, et al. Electricity-powered cryptic CO_2_ fixation pathway in heterotrophic *Shewanella oneidensis* for acetate synthesis[J]. Bioresource Technology, 2025,426:132324.

[9] Wu H, Ding L, Wang H, et al. In-situ biogenic FeS boosted acetate accumulation through CO2 capture and valorization using microbial electrosynthesis (MES)[J]. Chemical Engineering Journal, 2024,493:152563.
